# Supplementary material for: Data-driven analysis to understand long COVID using electronic health records from the RECOVER initiative
Source: Nat Commun. 2023 Apr 7;14:1948. doi: 10.1038/s41467-023-37653-z (PMC10080528; doi:10.1038/s41467-023-37653-z)
Supplement: Supplementary file 1 — Supplementary Information [file 41467_2023_37653_MOESM1_ESM.pdf]

Contents

Supplementary Fig. 1. Temporal trends of the lab-confirmed monthly new SARS-CoV-2 cases per 10,000 patients in the INSIGHT and OneFlorida+ cohorts, March 2020 to November 2021. 6

Supplementary Fig. 2. Stratified analysis of adjusted excess burden (adjusted excess cumulative incidence per 1,000 patients) of post-acute sequelae of SARS-CoV-2 infection (PASC) over different subgroups, the OneFlorida+ cohort, from March 2020 to November 2021. Subgroups were stratified by acute severity status, age groups, gender, race groups, and baseline pre-existing conditions. Different color panels represent different organ systems, including (from top to bottom): the nervous system, skin, respiratory system, circulatory system, and other signs. CAD, coronary artery disease; CKD, chronic kidney disease; CPD, chronic pulmonary disease; T2D, diabetes type 2; Healthy: no documented pre-existing conditions and no PASC-like symptoms at baseline. Two ICD-10 diagnosis codes B948 (sequelae of other specified infectious and parasitic diseases) and U099 (post-COVID-19 condition, unspecified) were also used to compare general post-acute sequelae of SARS-CoV-2 infection in different groups. The conditions with their aHRs' P-value $< 8.39 \times 10-5$(the Bonferroni-corrected significance threshold) were highlighted in red squares. The fraction of the subgroup population was shown at the top........ 7

Supplementary Fig. 3. Comparison of Adjusted hazard ratio of identified incident medications for PASC in the OneFlorida+ cohort versus INSIGHT. The 95% confidence intervals (CI) were reported. The aHR's P-value $< 8.39 \times 10-5$ (the Bonferroni-corrected threshold) was used for selecting significant medications. The conditions also replicated in the INSIGHT were marked by ‡ symbols. The color panels represent different organ systems, including (from top to bottom): the respiratory system, endocrine and metabolic, and other general signs. The aHR and its P-value were calculated by the Cox proportional hazard model and the Wald Chi-Square test. 8

Supplementary Fig. 4. Adjusted cumulative incidence (per 1,000 patients) of post-acute sequelae of SARS-CoV-2 infection (PASC) over different subgroups, the INSIGHT cohort, SARS-CoV-2 infected patients, from March 2020 to November 2021. Subgroups were stratified by their acute severity status, age groups, gender, race groups, and baseline pre-existing conditions. Different color panels represent different organ systems. CAD, coronary artery disease; CKD, chronic kidney disease; CPD, chronic pulmonary disease; T2D, diabetes type 2; Healthy: no documented pre-existing conditions and no PASC-like symptoms at baseline. Two ICD-10 diagnosis codes B948 (sequelae of other specified infectious and parasitic diseases) and U099 (post-COVID-19 condition, unspecified) were also used to compare general post-acute sequelae of SARS-CoV-2 infection in different groups. The PASC conditions also identified in OneFlorida+ were marked by ‡ symbols. The conditions with their aHRs' P-value $< 8.39 \times 10-5$(the Bonferroni-corrected significance threshold) were highlighted in red squares. The fraction of the subgroup population was shown at the top. 10

Supplementary Fig. 5. Adjusted cumulative incidence (per 1,000 patients) of post-acute sequelae of SARS-CoV-2 infection (PASC) over different subgroups, the OneFlorida+ cohort, SARS-CoV-2 infected patients, from March 2020 to November 2021. Subgroups were stratified by their acute severity status, age groups, gender, race groups, and baseline pre-existing conditions. Different color panels represent different organ systems, including (from top to bottom): the nervous system, skin, respiratory system, circulatory system, and general signs. CAD, coronary artery disease; CKD, chronic kidney disease; CPD, chronic pulmonary disease; T2D, diabetes type 2; Healthy: no documented pre-existing conditions and no PASC-like symptoms at baseline. Two ICD-10 diagnosis codes B948 (sequelae of other specified infectious and parasitic diseases) and U099 (post-COVID-19 condition, unspecified) were also used to compare general post-acute sequelae of SARS-CoV-2 infection in different groups. The conditions with their aHRs' P-value$< 8.39 \times 10 - 5$(the Bonferroni-corrected significance threshold) were highlighted in red squares. The fraction of the subgroup population was shown at the top. 11

Supplementary Fig. 6. Stratified analysis of adjusted excess burden (adjusted excess cumulative incidence per 1,000 patients) of post-acute sequelae of SARS-CoV-2 infection (PASC) over different periods, from March 2020 to November 2021. The ancestral strain wave was defined from March 1, 2020, to September 30, 2020, the Delta wave was defined from June 1, 2021, and November 30, 2021; and from October 1, 2020, to May 31, 2021, were Alpha and other variants. Different color panels represent different organ systems, including (from top to bottom): the nervous system, skin, respiratory system, circulatory system, and general signs. CAD, coronary artery disease; CKD, chronic kidney disease; CPD, chronic pulmonary disease; T2D, diabetes type 2; Healthy: no documented pre-existing conditions and no PASC-like symptoms at baseline. Two ICD-10 diagnosis codes B948 (sequelae of other specified infectious and parasitic diseases) and U099 (post-COVID-19 condition, unspecified) were also used to compare general post-acute sequelae of SARS-CoV-2 infection in different groups. The conditions with their aHRs' P-value$< 8.39 \times 10 - 5$(the Bonferroni-corrected significance threshold) were highlighted in red squares...............13

Supplementary Fig. 7. Additional post-acute sequelae of SARS-CoV-2 risks in the INSIGHT cohort versus in the OneFlorida+ cohort by using the Benjamini-Yekutieli method to control for false discovery rate, from March 2020 to November 2021. Incidence risk measured by adjusted hazard ratios and 95% confidence intervals (CI) were reported in the main panel. The adjusted cumulative incidences (CIF) per 1,000 patients in both the SARS-CoV-2 positive group and the negative group were also reported. The PASC conditions identified in both datasets were marked by ‡ symbols. The PASC outcomes were ascertained from day 30 after the SARS-CoV-2 infection and all the adjusted risk measures were computed 180 days after the SARS-CoV-2 infection. The threshold of the false discovery rate in the Benjamini-Yekutieli method is 0.05. GERD, gastroesophageal reflux disease...14

Supplementary Fig. 8. The post-acute sequelae of SARS-CoV-2 risks when adjusting for additional baseline vaccination status or not, the INSIGHT and OneFlorida+ cohorts, from March 2020 to November 2021. The baseline vaccination covariates include fully vaccinated, partially vaccinated, and no evidence of vaccination. We defined the fully

vaccinated as two shots of mRNA vaccine (Pfizer or Moderna) or one shot of J&J, see https://www.cdc.gov/coronavirus/2019-ncov/vaccines/stay-up-to-date.html. Adjusted hazard ratios (aHR) were compared when adjusting for these additional covariates (aHR w/ vax) versus not adjusting for these covariates in our primary analysis (aHR w/o vax). The PASC conditions identified in both datasets were marked by ‡ symbols. The 95% confidence intervals (CI) of aHR were reported. The color panels represent different organ systems, including (from top to bottom): the nervous system, skin, respiratory system, circulatory system, endocrine and metabolic, digestive system, genitourinary system, and other general signs. The PASC outcomes were ascertained from day 30 after the SARS-CoV-2 infection and all the adjusted risk measures were computed 180 days after the SARS-CoV-2 infection. 16

Supplementary Fig. 9. The post-acute sequelae of SARS-CoV-2 risks when adjusting for index day through Cubic B-Spline versus Categorical variables, the INSIGHT and OneFlorida+ cohorts, from March 2020 to November 2021. The index days since March 1st, 2020, were modeled by a) Cubic B-spline with five knots (namely, 7 spline basis functions of polynomial order 3) and b) categorizing days into five periods (March 2020 – June 2020, July 2020 – October 2020, November 2020 - February 2021, March 2021 – June 2021, July 2021 – November 2021). Adjusted hazard ratios (aHR) were compared when using the cubic B-spline method (aHR spline) versus the categorial method in the primary analysis (aHR category). The PASC conditions identified in both datasets were marked by ‡ symbols. The 95% confidence intervals (CI) of aHR were reported. The color panels represent different organ systems, including (from top to bottom): the nervous system, skin, respiratory system, circulatory system, endocrine and metabolic, digestive system, genitourinary system, and other general signs. The PASC outcomes were ascertained from day 30 after the SARS-CoV-2 infection and all the adjusted risk measures were computed 180 days after the SARS-CoV-2 infection. 18

Supplementary Fig. 10. The post-acute sequelae of SARS-CoV-2 risks when using non-linear PS modeling versus linear PS modeling, the INSIGHT and OneFlorida+ cohorts, from March 2020 to November 2021. Adjusted hazard ratios (aHR) were adjusted for a nonlinear PS modeling using the gradient boosting machine with decision tree base learners (aHR nonlinear) versus the linear PS modeling using regularized logistic regression in our primary analysis (aHR lin). The PASC conditions identified in both datasets were marked by ‡ symbols. The color panels represent different organ systems, including (from top to bottom): the nervous system, skin, respiratory system, circulatory system, endocrine and metabolic, digestive system, genitourinary system, and other general signs. The PASC outcomes were ascertained from day 30 after the SARS-CoV-2 infection and all the adjusted risk measures were computed 180 days after the SARS-CoV-2 infection. The 95% confidence intervals (CI) of aHR were reported. PS, propensity score. 20

Supplementary Fig. 11. The post-acute sequelae of SARS-CoV-2 risks when downsampling the INSIGHT cohort into 22,341 SARS-CoV-2 positive patients and 177,010 negative patients versus using original data, the INSIGHT and OneFlorida+ cohorts, from March 2020 to November 2021. The number of SARS-CoV2 positive (or negative) patients in the INSIGHT after downsampling is the same as the number of positive

(or negative) patients in the OneFlorida+. Adjusted hazard ratios (aHR) and 95% confidence intervals (CI) were compared using the downsampled cohorts (aHR sample) versus the original cohorts in our primary analysis (aHR all) for the INSIGHT cohort. The PASC conditions identified in both datasets were marked by ‡ symbols. The color panels represent different organ systems, including (from top to bottom): the nervous system, skin, respiratory system, circulatory system, endocrine and metabolic, digestive system, genitourinary system, and other general signs. The PASC outcomes were ascertained from day 30 after the SARS-CoV-2 infection and all the adjusted risk measures were computed 180 days after the SARS-CoV-2 infection. 22

Supplementary Fig. 12. Adjusted hazard ratios of post-acute sequelae of SARS-CoV-2 infection (PASC) over different subgroups, the INSIGHT cohort, from March 2020 to November 2021. Subgroups were stratified by their acute severity status, age groups, gender, race groups, and baseline pre-existing conditions. Different color panels represent different organ systems, including (from top to bottom): the nervous system, skin, respiratory system, circulatory system, blood-forming organs, endocrine and metabolic, digestive system, genitourinary system, and general signs. CAD, coronary artery disease; CKD, chronic kidney disease; CPD, chronic pulmonary disease; T2D, diabetes type 2; Healthy: no documented pre-existing conditions and no PASC-like symptoms at baseline. The PASC conditions also identified in OneFlorida+ were marked by ‡ symbols. The conditions with their aHRs' P-value$<\ 8.39 \times 10-5$(the Bonferroni-corrected significance threshold) were highlighted in red squares. The fraction of the subgroup population was shown at the top. 24

Supplementary Fig. 13. Adjusted hazard ratios of post-acute sequelae of SARS-CoV-2 infection (PASC) over different subgroups, the OneFlorida+ cohort, from March 2020 to November 2021. Subgroups were stratified by their acute severity status, age groups, gender, race groups, and baseline pre-existing conditions. Different color panels represent different organ systems, including (from top to bottom): the nervous system, skin, respiratory system, circulatory system, and general signs. CAD, coronary artery disease; CKD, chronic kidney disease; CPD, chronic pulmonary disease; T2D, diabetes type 2; Healthy: no documented pre-existing conditions and no PASC-like symptoms at baseline. The conditions with their aHRs' P-value$<\ 8.39 \times 10-5$(the Bonferroni-corrected significance threshold) were highlighted in red squares. The fraction of the subgroup population was shown at the top. 25

Supplementary Table 1. Results of negative outcome control in both the INSIGHT and OneFlorida+ cohorts, March 2020–November 2021. 26

Supplementary Table 2. Specifications of the high-throughput screening framework for identifying potential Post-Acute Sequelae of SARS-CoV-2 infected (PASC) using the INSIGHT Electronic Health Records in New York City and OneFlorida+ in Florida (March 2020 – November 2021). 28

Supplementary Table 3. Cross-validation algorithm tailored for our machine learning-based propensity score calculation for each emulated trial. 31

Supplementary Table 4. Baseline SARS-CoV-2 vaccination status recorded in the two EHR databases[a] 32

Supplementary Data 1. COVID-19 Phenotyping Lab LOINC codes and Diagnosis ICD10 codes (spreadsheet)..33

Supplementary Data 2. PASC Adult Diagnostic List for Screening (spreadsheet)................33

Supplementary Data 3. Baseline population characteristics with more comorbidities information, INSIGHT and OneFlorida+ cohorts, March 2020 to November 2021 (spreadsheet). 33

Supplementary Data 4. Characteristics of PASC-Specific Cohorts on INSIGHT, NYC, March 2020 to November 2021 (spreadsheet). 33

Supplementary Data 5. Characteristics of PASC-Specific Cohorts on OneFlorida, Florida, March 2020 to November 2021 (spreadsheet)...33

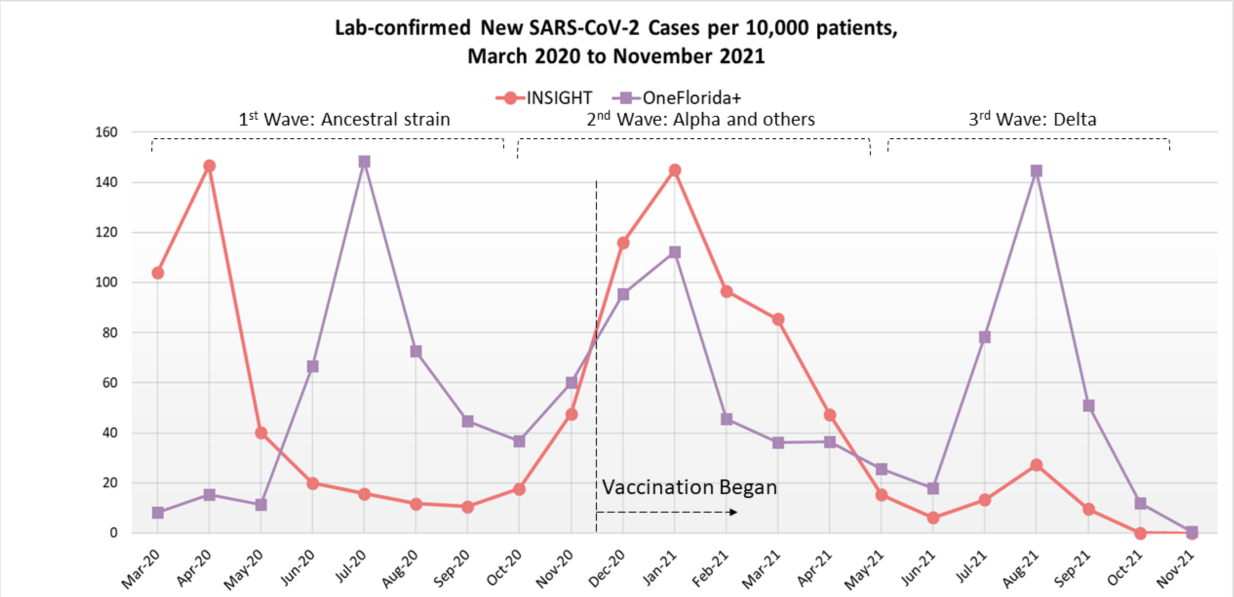

	Mar-20	Apr-20	May-20	Jun-20	Jul-20	Aug-20	Sep-20	Oct-20	Nov-20	Dec-20	Jan-21	Feb-21	Mar-21	Apr-21	May-21	Jun-21	Jul-21	Aug-21	Sep-21	Oct-21	Nov-21
INSIGHT	103.9	146.7	40.2	20.0	15.8	11.7	10.6	17.8	47.6	115.9	144.9	96.6	85.4	47.2	15.4	6.2	13.3	27.2	9.6	0.1	0.0
OneFlorida+	8.4	15.5	11.3	66.7	148.5	72.6	44.7	36.9	60.2	95.5	112.4	45.7	36.1	36.6	25.5	18.0	78.3	144.7	50.9	11.8	0.5

Supplementary Fig. 1. Temporal trends of the lab-confirmed monthly new SARS-CoV-2 cases per 10,000 patients in the INSIGHT and OneFlorida+ cohorts, March 2020 to November 2021.

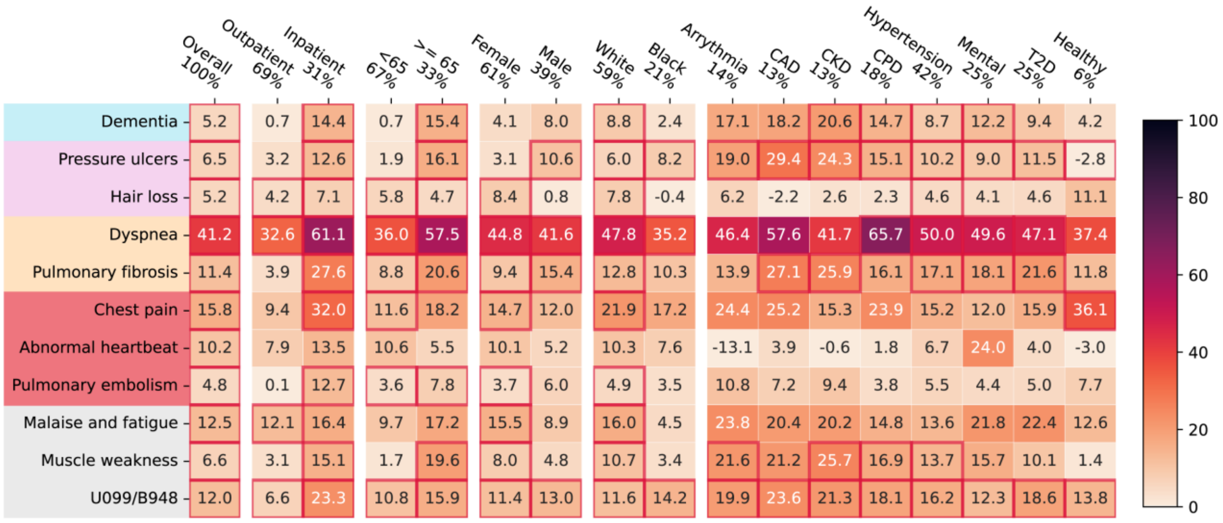

Supplementary Fig. 2. Stratified analysis of adjusted excess burden (adjusted excess cumulative incidence per 1,000 patients) of post-acute sequelae of SARS-CoV-2 infection (PASC) over different subgroups, the OneFlorida+ cohort, from March 2020 to November 2021. Subgroups were stratified by acute severity status, age groups, gender, race groups, and baseline pre-existing conditions. Different color panels represent different organ systems, including (from top to bottom): the nervous system, skin, respiratory system, circulatory system, and other signs. CAD, coronary artery disease; CKD, chronic kidney disease; CPD, chronic pulmonary disease; T2D, diabetes type 2; Healthy: no documented pre-existing conditions and no PASC-like symptoms at baseline. Two ICD-10 diagnosis codes B948 (sequelae of other specified infectious and parasitic diseases) and U099 (post-COVID-19 condition, unspecified) were also used to compare general post-acute sequelae of SARS-CoV-2 infection in different groups. The conditions with their aHRs' P-value $< 8.39 \times 10^{-5}$ (the Bonferroni-corrected significance threshold) were highlighted in red squares. The fraction of the subgroup population was shown at the top.

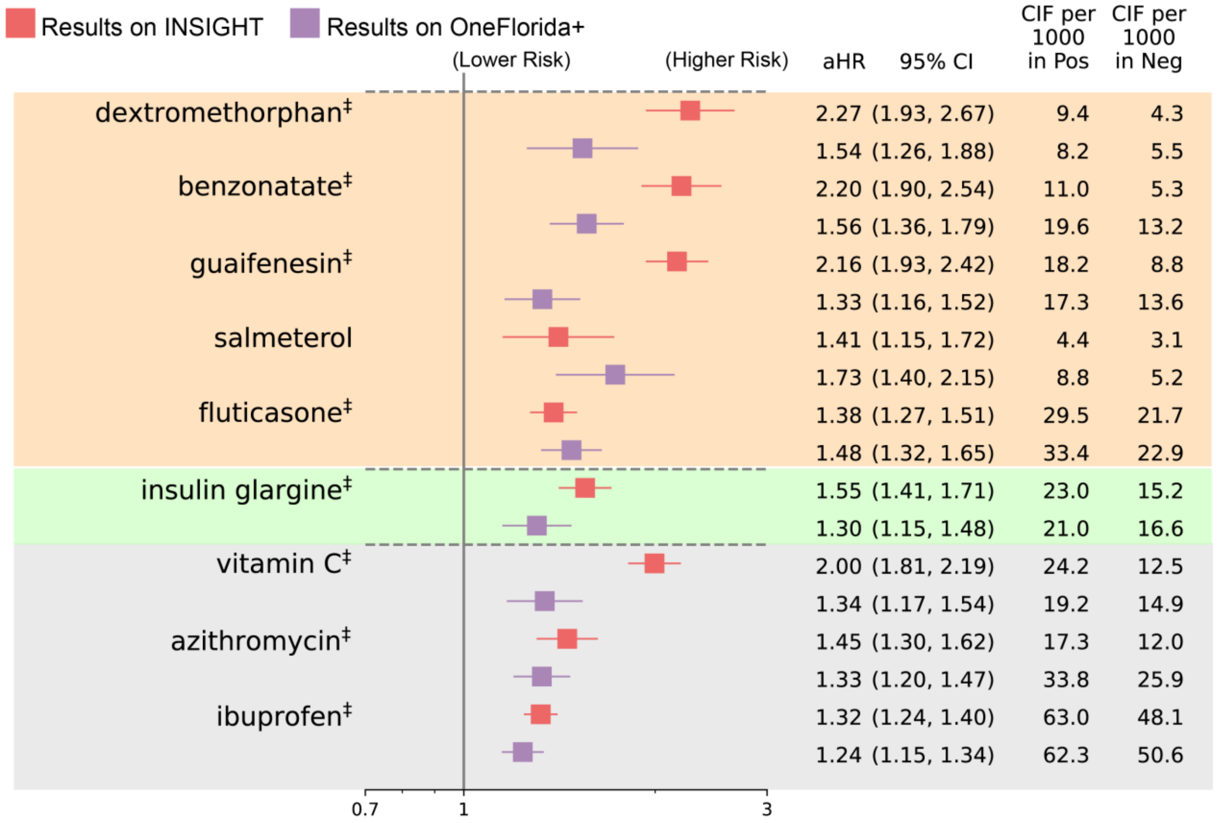

Supplementary Fig. 3. Comparison of Adjusted hazard ratio of identified incident medications for PASC in the OneFlorida+ cohort versus INSIGHT. The 95% confidence intervals (CI) were reported. The aHR's P-value < 8.39×10^{-5} (the Bonferroni-corrected threshold) was used for selecting significant medications. The conditions also replicated in the INSIGHT were marked by ‡ symbols. The color panels represent different organ systems, including (from top to bottom): the respiratory system, endocrine and metabolic, and other general signs. The aHR and its P-value were calculated by the Cox proportional hazard model and the Wald Chi-Square test.

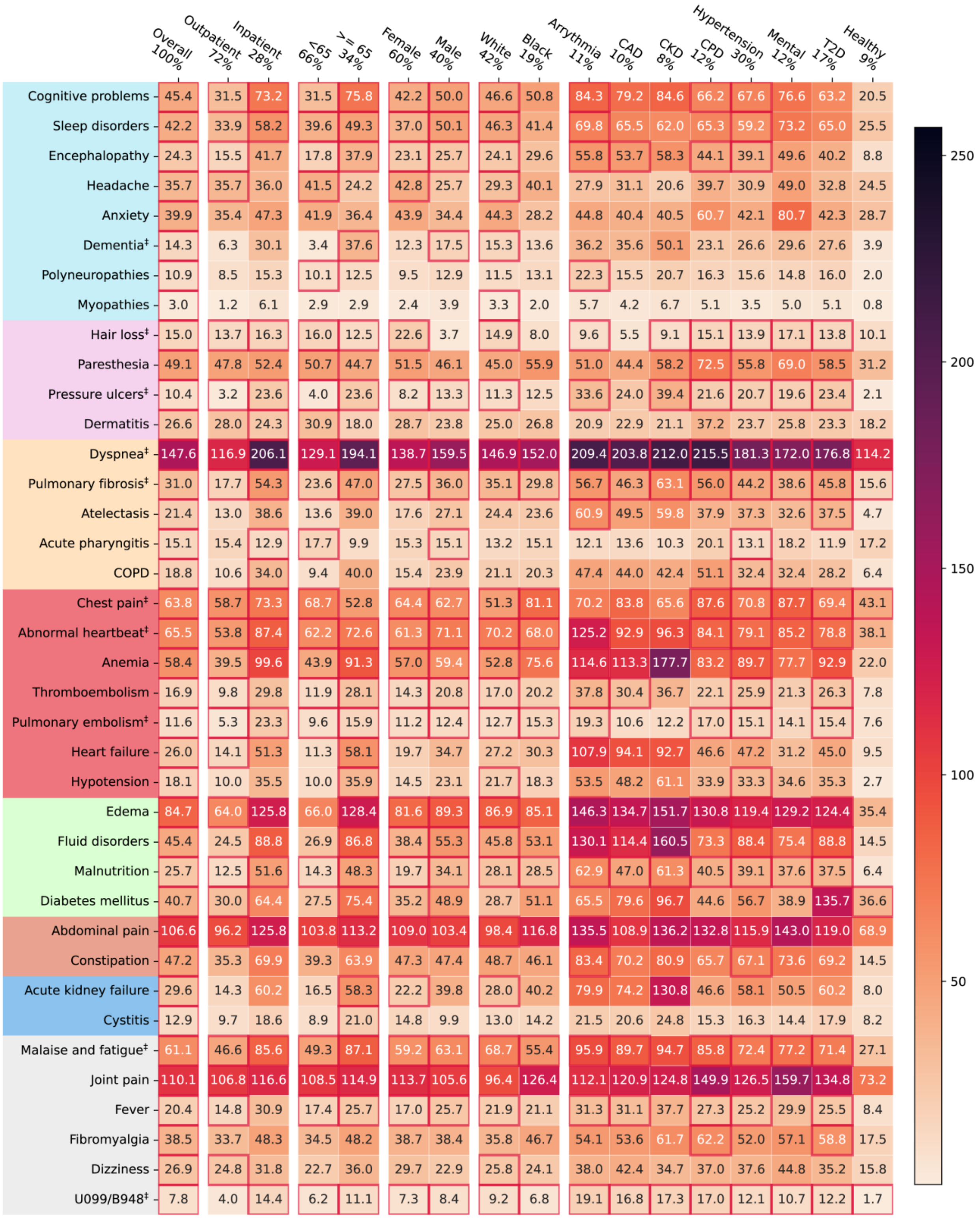

Overall 100%
Outpatient 72%
Inpatient 28%
<65 66%
>= 65 34%
Female 60%
Male 40%
White 42%
Black 19%
Arrythmia 11%
CAD 10%
CKD 8%
CPD 12%
Hypertension 30%
Mental 12%
T2D 17%
Healthy 9%
Cognitive problems 45.4 31.5 73.2 31.5 75.8 42.2 50.0 46.6 50.8 84.3 79.2 84.6 66.2 67.6 76.6 63.2 20.5
Sleep disorders 42.2 33.9 58.2 39.6 49.3 37.0 50.1 46.3 41.4 69.8 65.5 62.0 65.3 59.2 73.2 65.0 25.5
Encephalopathy 24.3 15.5 41.7 17.8 37.9 23.1 25.7 24.1 29.6 55.8 53.7 58.3 44.1 39.1 49.6 40.2 8.8
Headache 35.7 35.7 36.0 41.5 24.2 42.8 25.7 29.3 40.1 27.9 31.1 20.6 39.7 30.9 49.0 32.8 24.5
Anxiety 39.9 35.4 47.3 41.9 36.4 43.9 34.4 44.3 28.2 44.8 40.4 40.5 60.7 42.1 80.7 42.3 28.7
Dementia‡ 14.3 6.3 30.1 3.4 37.6 12.3 17.5 15.3 13.6 36.2 35.6 50.1 23.1 26.6 29.6 27.6 3.9
Polyneuropathies 10.9 8.5 15.3 10.1 12.5 9.5 12.9 11.5 13.1 22.3 15.5 20.7 16.3 15.6 14.8 16.0 2.0
Myopathies 3.0 1.2 6.1 2.9 2.9 2.4 3.9 3.3 2.0 5.7 4.2 6.7 5.1 3.5 5.0 5.1 0.8
Hair loss‡ 15.0 13.7 16.3 16.0 12.5 22.6 3.7 14.9 8.0 9.6 5.5 9.1 15.1 13.9 17.1 13.8 10.1
Paresthesia 49.1 47.8 52.4 50.7 44.7 51.5 46.1 45.0 55.9 51.0 44.4 58.2 72.5 55.8 69.0 58.5 31.2
Pressure ulcers‡ 10.4 3.2 23.6 4.0 23.6 8.2 13.3 11.3 12.5 33.6 24.0 39.4 21.6 20.7 19.6 23.4 2.1
Dermatitis 26.6 28.0 24.3 30.9 18.0 28.7 23.8 25.0 26.8 20.9 22.9 21.1 37.2 23.7 25.8 23.3 18.2
Dyspnea‡ 147.6 116.9 206.1 129.1 194.1 138.7 159.5 146.9 152.0 209.4 203.8 212.0 215.5 181.3 172.0 176.8 114.2
Pulmonary fibrosis‡ 31.0 17.7 54.3 23.6 47.0 27.5 36.0 35.1 29.8 56.7 46.3 63.1 56.0 44.2 38.6 45.8 15.6
Atelectasis 21.4 13.0 38.6 13.6 39.0 17.6 27.1 24.4 23.6 60.9 49.5 59.8 37.9 37.3 32.6 37.5 4.7
Acute pharyngitis 15.1 15.4 12.9 17.7 9.9 15.3 15.1 13.2 15.1 12.1 13.6 10.3 20.1 13.1 18.2 11.9 17.2
COPD 18.8 10.6 34.0 9.4 40.0 15.4 23.9 21.1 20.3 47.4 44.0 42.4 51.1 32.4 32.4 28.2 6.4
Chest pain‡ 63.8 58.7 73.3 68.7 52.8 64.4 62.7 51.3 81.1 70.2 83.8 65.6 87.6 70.8 87.7 69.4 43.1
Abnormal heartbeat‡ 65.5 53.8 87.4 62.2 72.6 61.3 71.1 70.2 68.0 125.2 92.9 96.3 84.1 79.1 85.2 78.8 38.1
Anemia 58.4 39.5 99.6 43.9 91.3 57.0 59.4 52.8 75.6 114.6 113.3 177.7 83.2 89.7 77.7 92.9 22.0
Thromboembolism 16.9 9.8 29.8 11.9 28.1 14.3 20.8 17.0 20.2 37.8 30.4 36.7 22.1 25.9 21.3 26.3 7.8
Pulmonary embolism‡ 11.6 5.3 23.3 9.6 15.9 11.2 12.4 12.7 15.3 19.3 10.6 12.2 17.0 15.1 14.1 15.4 7.6
Heart failure 26.0 14.1 51.3 11.3 58.1 19.7 34.7 27.2 30.3 107.9 94.1 92.7 46.6 47.2 31.2 45.0 9.5
Hypotension 18.1 10.0 35.5 10.0 35.9 14.5 23.1 21.7 18.3 53.5 48.2 61.1 33.9 33.3 34.6 35.3 2.7
Edema 84.7 64.0 125.8 66.0 128.4 81.6 89.3 86.9 85.1 146.3 134.7 151.7 130.8 119.4 129.2 124.4 35.4
Fluid disorders 45.4 24.5 88.8 26.9 86.8 38.4 55.3 45.8 53.1 130.1 114.4 160.5 73.3 88.4 75.4 88.8 14.5
Malnutrition 25.7 12.5 51.6 14.3 48.3 19.7 34.1 28.1 28.5 62.9 47.0 61.3 40.5 39.1 37.6 37.5 6.4
Diabetes mellitus 40.7 30.0 64.4 27.5 75.4 35.2 48.9 28.7 51.1 65.5 79.6 96.7 44.6 56.7 38.9 135.7 36.6
Abdominal pain 106.6 96.2 125.8 103.8 113.2 109.0 103.4 98.4 116.8 135.5 108.9 136.2 132.8 115.9 143.0 119.0 68.9
Constipation 47.2 35.3 69.9 39.3 63.9 47.3 47.4 48.7 46.1 83.4 70.2 80.9 65.7 67.1 73.6 69.2 14.5
Acute kidney failure 29.6 14.3 60.2 16.5 58.3 22.2 39.8 28.0 40.2 79.9 74.2 130.8 46.6 58.1 50.5 60.2 8.0
Cystitis 12.9 9.7 18.6 8.9 21.0 14.8 9.9 13.0 14.2 21.5 20.6 24.8 15.3 16.3 14.4 17.9 8.2
Malaise and fatigue‡ 61.1 46.6 85.6 49.3 87.1 59.2 63.1 68.7 55.4 95.9 89.7 94.7 85.8 72.4 77.2 71.4 27.1
Joint pain 110.1 106.8 116.6 108.5 114.9 113.7 105.6 96.4 126.4 112.1 120.9 124.8 149.9 126.5 159.7 134.8 73.2
Fever 20.4 14.8 30.9 17.4 25.7 17.0 25.7 21.9 21.1 31.3 31.1 37.7 27.3 25.2 29.9 25.5 8.4
Fibromyalgia 38.5 33.7 48.3 34.5 48.2 38.7 38.4 35.8 46.7 54.1 53.6 61.7 62.2 52.0 57.1 58.8 17.5
Dizziness 26.9 24.8 31.8 22.7 36.0 29.7 22.9 25.8 24.1 38.0 42.4 34.7 37.0 37.6 44.8 35.2 15.8
U099/B948‡ 7.8 4.0 14.4 6.2 11.1 7.3 8.4 9.2 6.8 19.1 16.8 17.3 17.0 12.1 10.7 12.2 1.7
250
200
150
100
50

Supplementary Fig. 4. Adjusted cumulative incidence (per 1,000 patients) of post-acute sequelae of SARS-CoV-2 infection (PASC) over different subgroups, the INSIGHT cohort, SARS-CoV-2 infected patients, from March 2020 to November 2021. Subgroups were stratified by their acute severity status, age groups, gender, race groups, and baseline pre-existing conditions. Different color panels represent different organ systems. CAD, coronary artery disease; CKD, chronic kidney disease; CPD, chronic pulmonary disease; T2D, diabetes type 2; Healthy: no documented pre-existing conditions and no PASC-like symptoms at baseline. Two ICD-10 diagnosis codes B948 (sequelae of other specified infectious and parasitic diseases) and U099 (post-COVID-19 condition, unspecified) were also used to compare general post-acute sequelae of SARS-CoV-2 infection in different groups. The PASC conditions also identified in OneFlorida+ were marked by ‡ symbols. The conditions with their aHRs' P-value $< 8.39 \times 10^{-5}$(the Bonferroni-corrected significance threshold) were highlighted in red squares. The fraction of the subgroup population was shown at the top.

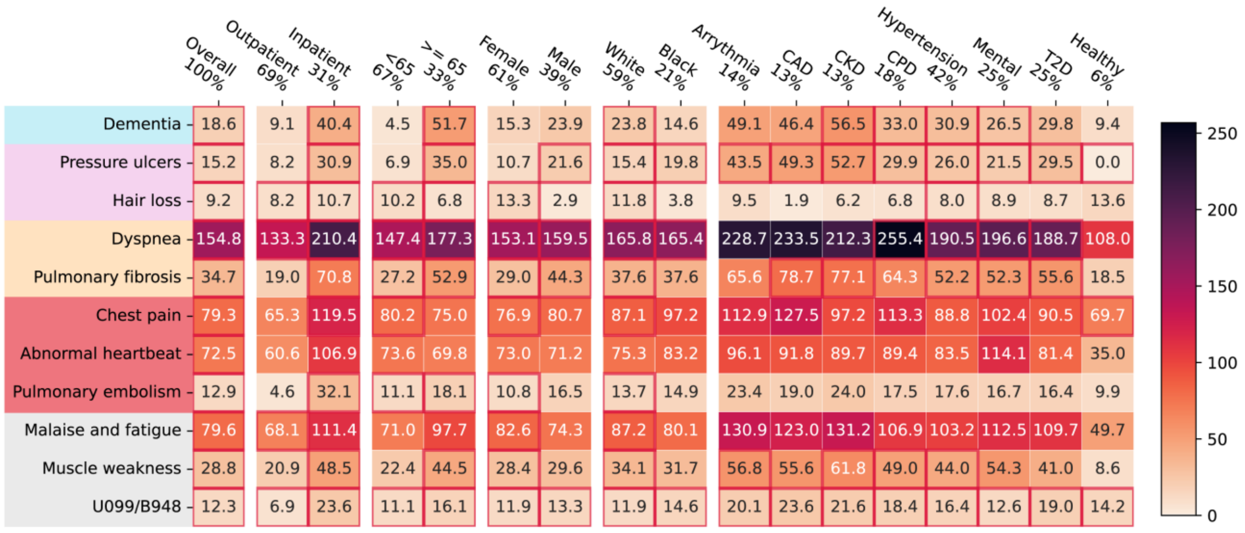

Supplementary Fig. 5. Adjusted cumulative incidence (per 1,000 patients) of post-acute sequelae of SARS-CoV-2 infection (PASC) over different subgroups, the OneFlorida+ cohort, SARS-CoV-2 infected patients, from March 2020 to November 2021. Subgroups were stratified by their acute severity status, age groups, gender, race groups, and baseline pre-existing conditions. Different color panels represent different organ systems, including (from top to bottom): the nervous system, skin, respiratory system, circulatory system, and general signs. CAD, coronary artery disease; CKD, chronic kidney disease; CPD, chronic pulmonary disease; T2D, diabetes type 2; Healthy: no documented pre-existing conditions and no PASC-like symptoms at baseline. Two ICD-10 diagnosis codes B948 (sequelae of other specified infectious and parasitic diseases) and U099 (post-COVID-19 condition, unspecified) were also used to compare general post-acute sequelae of SARS-CoV-2 infection in different groups. The conditions with their aHRs' P-value$< 8.39 \times 10^{-5}$(the Bonferroni-corrected significance threshold) were highlighted in red squares. The fraction of the subgroup population was shown at the top.

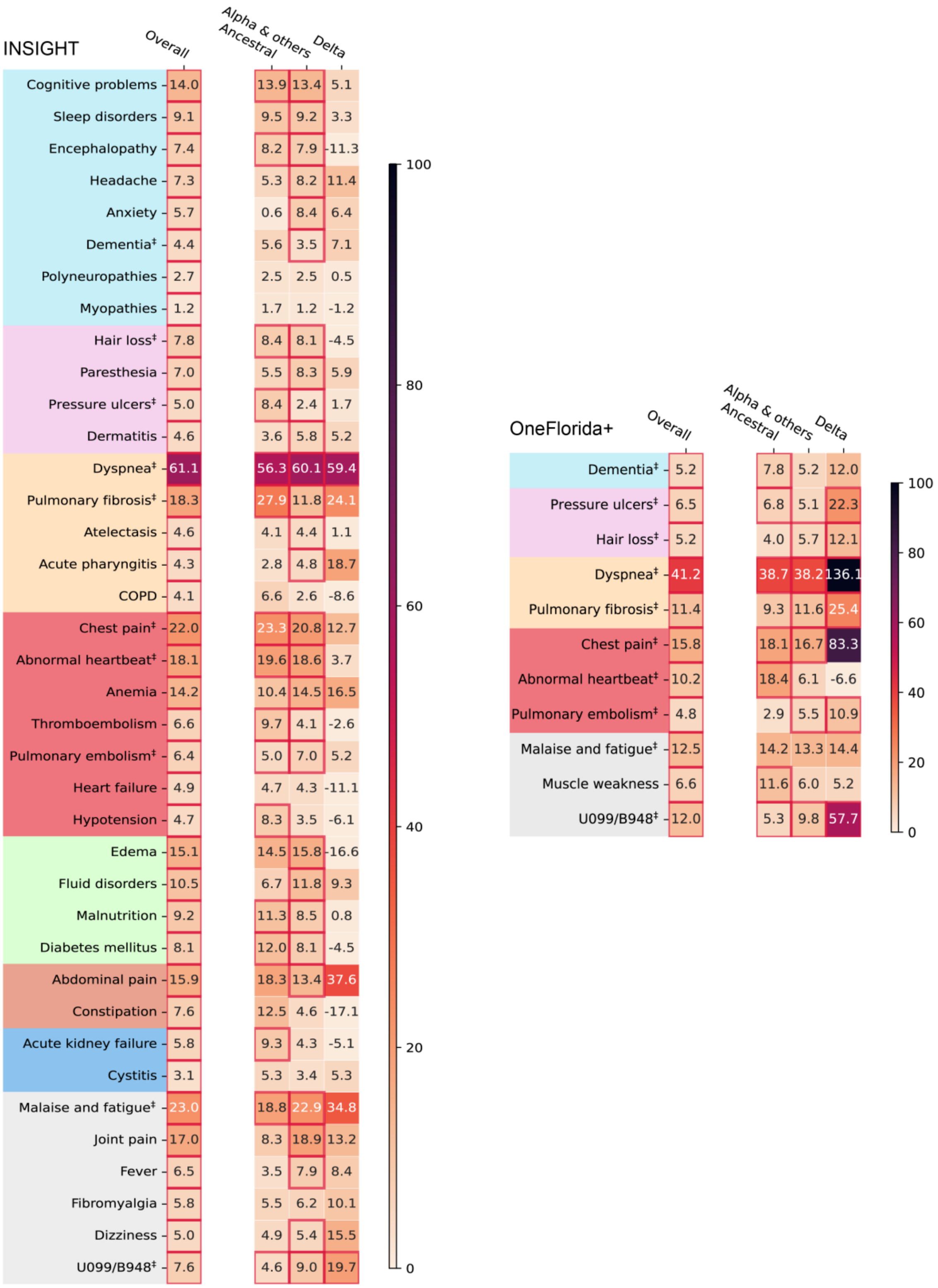
INSIGHT
Overall
Ancestral
Alpha & others
Delta
Cognitive problems 14.0 13.9 13.4 5.1
Sleep disorders 9.1 9.5 9.2 3.3
Encephalopathy 7.4 8.2 7.9 -11.3
Headache 7.3 5.3 8.2 11.4
Anxiety 5.7 0.6 8.4 6.4
Dementia‡ 4.4 5.6 3.5 7.1
Polyneuropathies 2.7 2.5 2.5 0.5
Myopathies 1.2 1.7 1.2 -1.2
Hair loss‡ 7.8 8.4 8.1 -4.5
Paresthesia 7.0 5.5 8.3 5.9
Pressure ulcers‡ 5.0 8.4 2.4 1.7
Dermatitis 4.6 3.6 5.8 5.2
Dyspnea‡ 61.1 56.3 60.1 59.4
Pulmonary fibrosis‡ 18.3 27.9 11.8 24.1
Atelectasis 4.6 4.1 4.4 1.1
Acute pharyngitis 4.3 2.8 4.8 18.7
COPD 4.1 6.6 2.6 -8.6
Chest pain‡ 22.0 23.3 20.8 12.7
Abnormal heartbeat‡ 18.1 19.6 18.6 3.7
Anemia 14.2 10.4 14.5 16.5
Thromboembolism 6.6 9.7 4.1 -2.6
Pulmonary embolism‡ 6.4 5.0 7.0 5.2
Heart failure 4.9 4.7 4.3 -11.1
Hypotension 4.7 8.3 3.5 -6.1
Edema 15.1 14.5 15.8 -16.6
Fluid disorders 10.5 6.7 11.8 9.3
Malnutrition 9.2 11.3 8.5 0.8
Diabetes mellitus 8.1 12.0 8.1 -4.5
Abdominal pain 15.9 18.3 13.4 37.6
Constipation 7.6 12.5 4.6 -17.1
Acute kidney failure 5.8 9.3 4.3 -5.1
Cystitis 3.1 5.3 3.4 5.3
Malaise and fatigue‡ 23.0 18.8 22.9 34.8
Joint pain 17.0 8.3 18.9 13.2
Fever 6.5 3.5 7.9 8.4
Fibromyalgia 5.8 5.5 6.2 10.1
Dizziness 5.0 4.9 5.4 15.5
U099/B948‡ 7.6 4.6 9.0 19.7
100
80
60
40
20
0
OneFlorida+
Overall
Ancestral
Alpha & others
Delta
Dementia‡ 5.2 7.8 5.2 12.0
Pressure ulcers‡ 6.5 6.8 5.1 22.3
Hair loss‡ 5.2 4.0 5.7 12.1
Dyspnea‡ 41.2 38.7 38.2 136.1
Pulmonary fibrosis‡ 11.4 9.3 11.6 25.4
Chest pain‡ 15.8 18.1 16.7 83.3
Abnormal heartbeat‡ 10.2 18.4 6.1 -6.6
Pulmonary embolism‡ 4.8 2.9 5.5 10.9
Malaise and fatigue‡ 12.5 14.2 13.3 14.4
Muscle weakness 6.6 11.6 6.0 5.2
U099/B948‡ 12.0 5.3 9.8 57.7
100
80
60
40
20
0

Supplementary Fig. 6. Stratified analysis of adjusted excess burden (adjusted excess cumulative incidence per 1,000 patients) of post-acute sequelae of SARS-CoV-2 infection (PASC) over different periods, from March 2020 to November 2021. The ancestral strain wave was defined from March 1, 2020, to September 30, 2020, the Delta wave was defined from June 1, 2021, and November 30, 2021; and from October 1, 2020, to May 31, 2021, were Alpha and other variants. Different color panels represent different organ systems, including (from top to bottom): the nervous system, skin, respiratory system, circulatory system, and general signs. CAD, coronary artery disease; CKD, chronic kidney disease; CPD, chronic pulmonary disease; T2D, diabetes type 2; Healthy: no documented pre-existing conditions and no PASC-like symptoms at baseline. Two ICD-10 diagnosis codes B948 (sequelae of other specified infectious and parasitic diseases) and U099 (post-COVID-19 condition, unspecified) were also used to compare general post-acute sequelae of SARS-CoV-2 infection in different groups. The conditions with their aHRs' P-value $< 8.39 \times 10^{-5}$(the Bonferroni-corrected significance threshold) were highlighted in red squares.

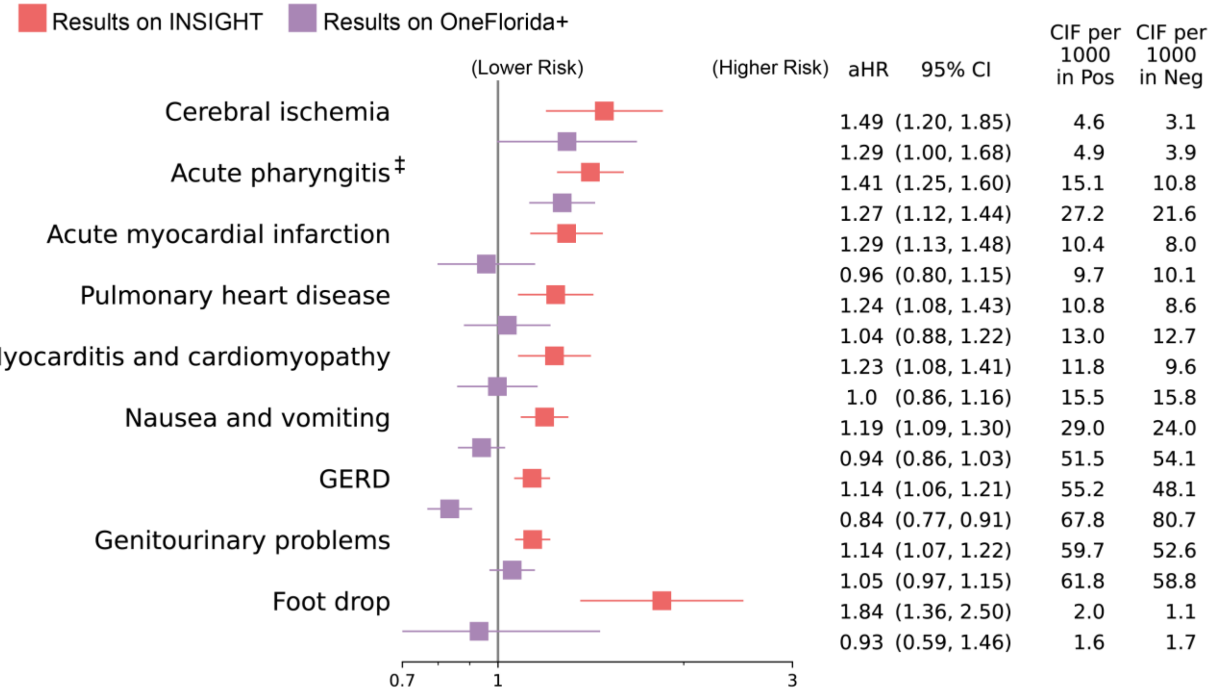

Supplementary Fig. 7. Additional post-acute sequelae of SARS-CoV-2 risks in the INSIGHT cohort versus in the OneFlorida+ cohort by using the Benjamini-Yekutieli method to control for false discovery rate, from March 2020 to November 2021. Incidence risk measured by adjusted hazard ratios and 95% confidence intervals (CI) were reported in the main panel. The adjusted cumulative incidences (CIF) per 1,000 patients in both the SARS-CoV-2 positive group and the negative group were also reported. The PASC conditions identified in both datasets were marked by ‡ symbols. The PASC outcomes were ascertained from day 30 after the SARS-CoV-2 infection and all the adjusted risk measures were computed 180 days after the SARS-CoV-2 infection. The threshold of the false discovery rate in the Benjamini-Yekutieli method is 0.05. GERD, gastroesophageal reflux disease.

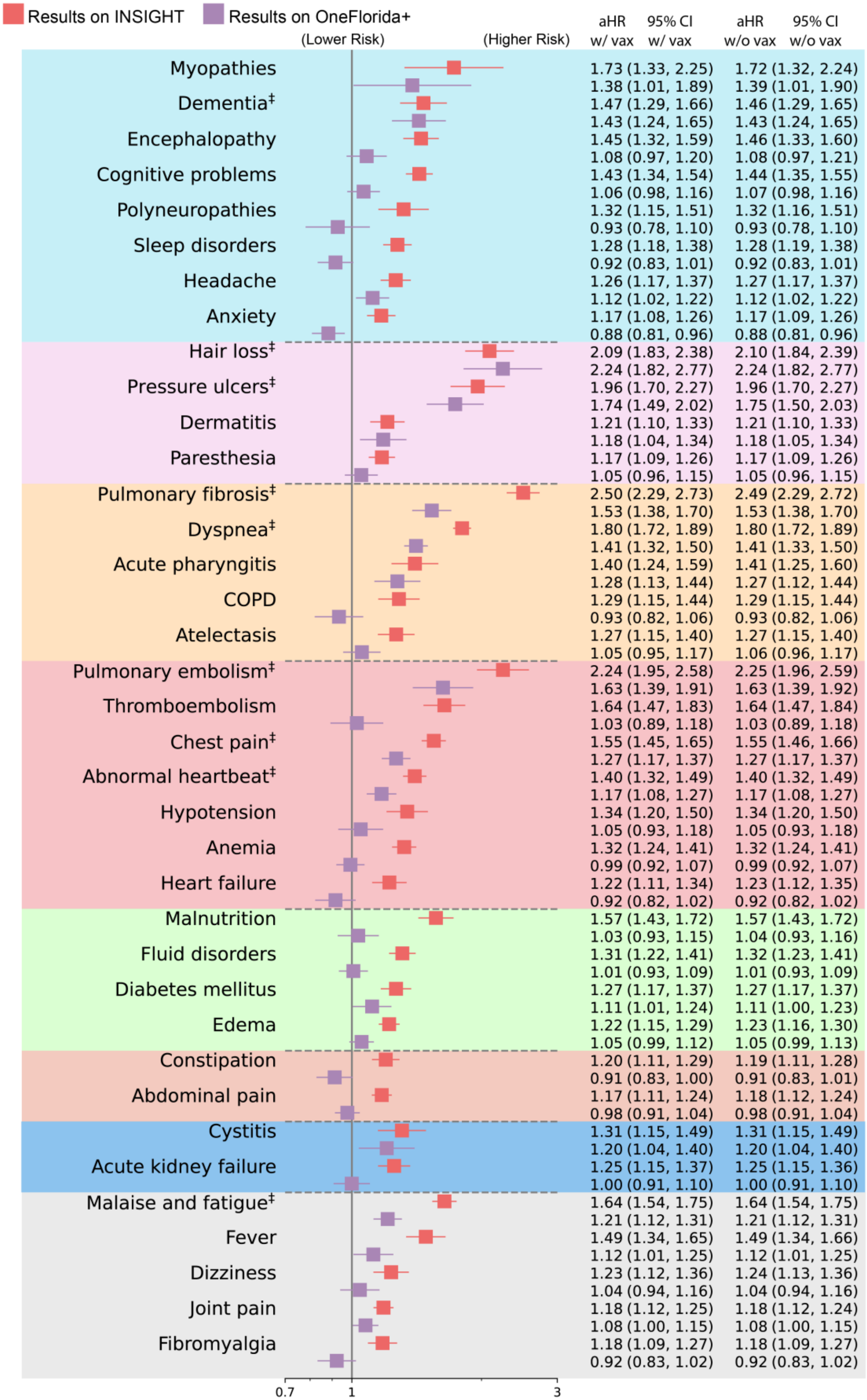

Outcome	Cohort	aHR w/ vax	95% CI w/ vax	aHR w/o vax	95% CI w/o vax
Myopathies	INSIGHT	1.73	(1.33, 2.25)	1.72	(1.32, 2.24)
	OneFlorida+	1.38	(1.01, 1.89)	1.39	(1.01, 1.90)
Dementia‡	INSIGHT	1.47	(1.29, 1.66)	1.46	(1.29, 1.65)
	OneFlorida+	1.43	(1.24, 1.65)	1.43	(1.24, 1.65)
Encephalopathy	INSIGHT	1.45	(1.32, 1.59)	1.46	(1.33, 1.60)
	OneFlorida+	1.08	(0.97, 1.20)	1.08	(0.97, 1.21)
Cognitive problems	INSIGHT	1.43	(1.34, 1.54)	1.44	(1.35, 1.55)
	OneFlorida+	1.06	(0.98, 1.16)	1.07	(0.98, 1.16)
Polyneuropathies	INSIGHT	1.32	(1.15, 1.51)	1.32	(1.16, 1.51)
	OneFlorida+	0.93	(0.78, 1.10)	0.93	(0.78, 1.10)
Sleep disorders	INSIGHT	1.28	(1.18, 1.38)	1.28	(1.19, 1.38)
	OneFlorida+	0.92	(0.83, 1.01)	0.92	(0.83, 1.01)
Headache	INSIGHT	1.26	(1.17, 1.37)	1.27	(1.17, 1.37)
	OneFlorida+	1.12	(1.02, 1.22)	1.12	(1.02, 1.22)
Anxiety	INSIGHT	1.17	(1.08, 1.26)	1.17	(1.09, 1.26)
	OneFlorida+	0.88	(0.81, 0.96)	0.88	(0.81, 0.96)
Hair loss‡	INSIGHT	2.09	(1.83, 2.38)	2.10	(1.84, 2.39)
	OneFlorida+	2.24	(1.82, 2.77)	2.24	(1.82, 2.77)
Pressure ulcers‡	INSIGHT	1.96	(1.70, 2.27)	1.96	(1.70, 2.27)
	OneFlorida+	1.74	(1.49, 2.02)	1.75	(1.50, 2.03)
Dermatitis	INSIGHT	1.21	(1.10, 1.33)	1.21	(1.10, 1.33)
	OneFlorida+	1.18	(1.04, 1.34)	1.18	(1.05, 1.34)
Paresthesia	INSIGHT	1.17	(1.09, 1.26)	1.17	(1.09, 1.26)
	OneFlorida+	1.05	(0.96, 1.15)	1.05	(0.96, 1.15)
Pulmonary fibrosis‡	INSIGHT	2.50	(2.29, 2.73)	2.49	(2.29, 2.72)
	OneFlorida+	1.53	(1.38, 1.70)	1.53	(1.38, 1.70)
Dyspnea‡	INSIGHT	1.80	(1.72, 1.89)	1.80	(1.72, 1.89)
	OneFlorida+	1.41	(1.32, 1.50)	1.41	(1.33, 1.50)
Acute pharyngitis	INSIGHT	1.40	(1.24, 1.59)	1.41	(1.25, 1.60)
	OneFlorida+	1.28	(1.13, 1.44)	1.27	(1.12, 1.44)
COPD	INSIGHT	1.29	(1.15, 1.44)	1.29	(1.15, 1.44)
	OneFlorida+	0.93	(0.82, 1.06)	0.93	(0.82, 1.06)
Atelectasis	INSIGHT	1.27	(1.15, 1.40)	1.27	(1.15, 1.40)
	OneFlorida+	1.05	(0.95, 1.17)	1.06	(0.96, 1.17)
Pulmonary embolism‡	INSIGHT	2.24	(1.95, 2.58)	2.25	(1.96, 2.59)
	OneFlorida+	1.63	(1.39, 1.91)	1.63	(1.39, 1.92)
Thromboembolism	INSIGHT	1.64	(1.47, 1.83)	1.64	(1.47, 1.84)
	OneFlorida+	1.03	(0.89, 1.18)	1.03	(0.89, 1.18)
Chest pain‡	INSIGHT	1.55	(1.45, 1.65)	1.55	(1.46, 1.66)
	OneFlorida+	1.27	(1.17, 1.37)	1.27	(1.17, 1.37)
Abnormal heartbeat‡	INSIGHT	1.40	(1.32, 1.49)	1.40	(1.32, 1.49)
	OneFlorida+	1.17	(1.08, 1.27)	1.17	(1.08, 1.27)
Hypotension	INSIGHT	1.34	(1.20, 1.50)	1.34	(1.20, 1.50)
	OneFlorida+	1.05	(0.93, 1.18)	1.05	(0.93, 1.18)
Anemia	INSIGHT	1.32	(1.24, 1.41)	1.32	(1.24, 1.41)
	OneFlorida+	0.99	(0.92, 1.07)	0.99	(0.92, 1.07)
Heart failure	INSIGHT	1.22	(1.11, 1.34)	1.23	(1.12, 1.35)
	OneFlorida+	0.92	(0.82, 1.02)	0.92	(0.82, 1.02)
Malnutrition	INSIGHT	1.57	(1.43, 1.72)	1.57	(1.43, 1.72)
	OneFlorida+	1.03	(0.93, 1.15)	1.04	(0.93, 1.16)
Fluid disorders	INSIGHT	1.31	(1.22, 1.41)	1.32	(1.23, 1.41)
	OneFlorida+	1.01	(0.93, 1.09)	1.01	(0.93, 1.09)
Diabetes mellitus	INSIGHT	1.27	(1.17, 1.37)	1.27	(1.17, 1.37)
	OneFlorida+	1.11	(1.01, 1.24)	1.11	(1.00, 1.23)
Edema	INSIGHT	1.22	(1.15, 1.29)	1.23	(1.16, 1.30)
	OneFlorida+	1.05	(0.99, 1.12)	1.05	(0.99, 1.13)
Constipation	INSIGHT	1.20	(1.11, 1.29)	1.19	(1.11, 1.28)
	OneFlorida+	0.91	(0.83, 1.00)	0.91	(0.83, 1.01)
Abdominal pain	INSIGHT	1.17	(1.11, 1.24)	1.18	(1.12, 1.24)
	OneFlorida+	0.98	(0.91, 1.04)	0.98	(0.91, 1.04)
Cystitis	INSIGHT	1.31	(1.15, 1.49)	1.31	(1.15, 1.49)
	OneFlorida+	1.20	(1.04, 1.40)	1.20	(1.04, 1.40)
Acute kidney failure	INSIGHT	1.25	(1.15, 1.37)	1.25	(1.15, 1.36)
	OneFlorida+	1.00	(0.91, 1.10)	1.00	(0.91, 1.10)
Malaise and fatigue‡	INSIGHT	1.64	(1.54, 1.75)	1.64	(1.54, 1.75)
	OneFlorida+	1.21	(1.12, 1.31)	1.21	(1.12, 1.31)
Fever	INSIGHT	1.49	(1.34, 1.65)	1.49	(1.34, 1.66)
	OneFlorida+	1.12	(1.01, 1.25)	1.12	(1.01, 1.25)
Dizziness	INSIGHT	1.23	(1.12, 1.36)	1.24	(1.13, 1.36)
	OneFlorida+	1.04	(0.94, 1.16)	1.04	(0.94, 1.16)
Joint pain	INSIGHT	1.18	(1.12, 1.25)	1.18	(1.12, 1.24)
	OneFlorida+	1.08	(1.00, 1.15)	1.08	(1.00, 1.15)
Fibromyalgia	INSIGHT	1.18	(1.09, 1.27)	1.18	(1.09, 1.27)
	OneFlorida+	0.92	(0.83, 1.02)	0.92	(0.83, 1.02)

Supplementary Fig. 8. The post-acute sequelae of SARS-CoV-2 risks when adjusting for additional baseline vaccination status or not, the INSIGHT and OneFlorida+ cohorts, from March 2020 to November 2021. The baseline vaccination covariates include fully vaccinated, partially vaccinated, and no evidence of vaccination. We defined the fully vaccinated as two shots of mRNA vaccine (Pfizer or Moderna) or one shot of J&J, see https://www.cdc.gov/coronavirus/2019-ncov/vaccines/stay-up-to-date.html. Adjusted hazard ratios (aHR) were compared when adjusting for these additional covariates (aHR w/ vax) versus not adjusting for these covariates in our primary analysis (aHR w/o vax). The PASC conditions identified in both datasets were marked by ‡ symbols. The 95% confidence intervals (CI) of aHR were reported. The color panels represent different organ systems, including (from top to bottom): the nervous system, skin, respiratory system, circulatory system, endocrine and metabolic, digestive system, genitourinary system, and other general signs. The PASC outcomes were ascertained from day 30 after the SARS-CoV-2 infection and all the adjusted risk measures were computed 180 days after the SARS-CoV-2 infection.

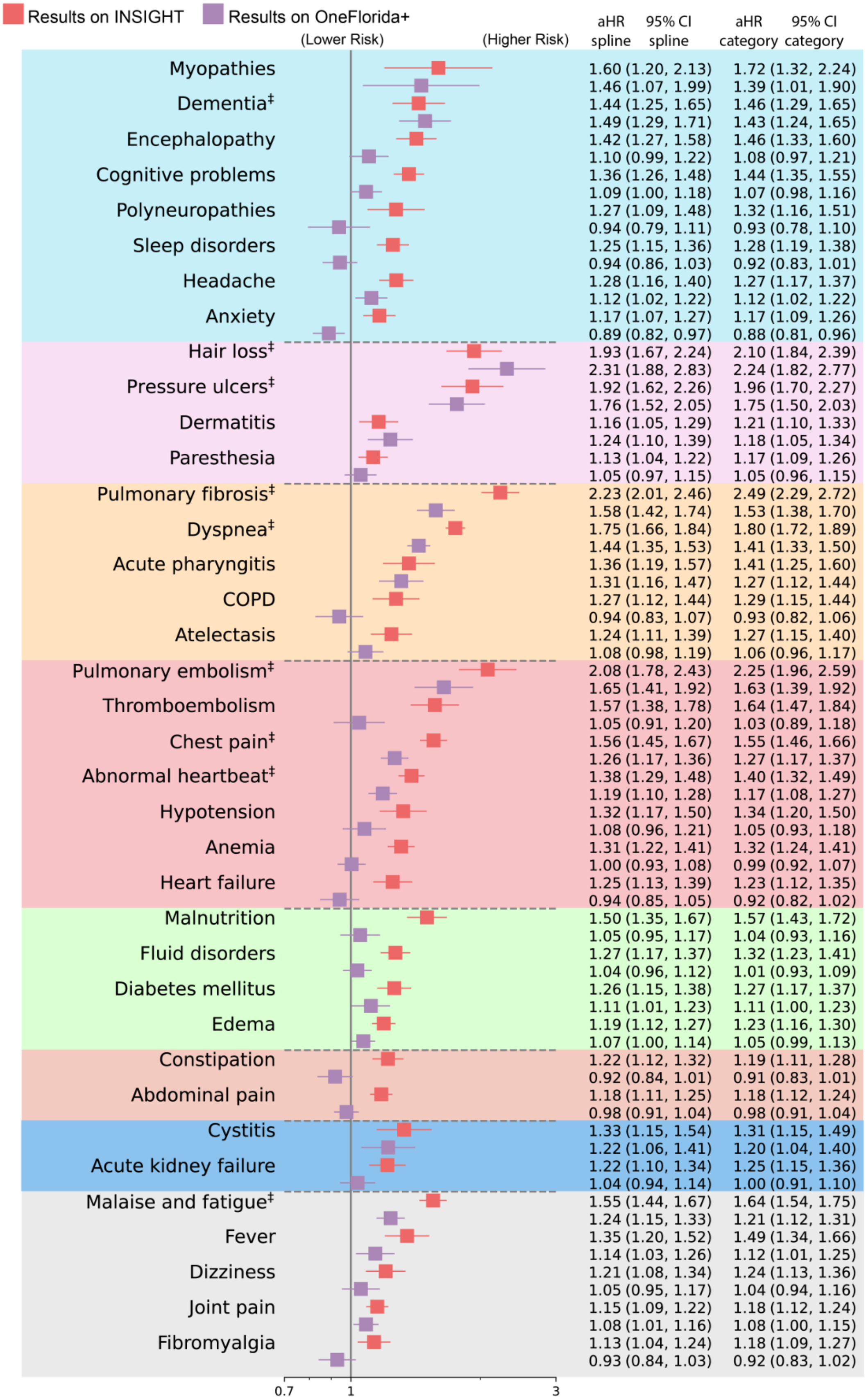
Results on INSIGHT
Results on OneFlorida+
(Lower Risk)
(Higher Risk)
aHR spline
95% CI spline
aHR category
95% CI category
Myopathies
1.60 (1.20, 2.13) 1.72 (1.32, 2.24)
1.46 (1.07, 1.99) 1.39 (1.01, 1.90)
Dementia‡
1.44 (1.25, 1.65) 1.46 (1.29, 1.65)
1.49 (1.29, 1.71) 1.43 (1.24, 1.65)
Encephalopathy
1.42 (1.27, 1.58) 1.46 (1.33, 1.60)
1.10 (0.99, 1.22) 1.08 (0.97, 1.21)
Cognitive problems
1.36 (1.26, 1.48) 1.44 (1.35, 1.55)
1.09 (1.00, 1.18) 1.07 (0.98, 1.16)
Polyneuropathies
1.27 (1.09, 1.48) 1.32 (1.16, 1.51)
0.94 (0.79, 1.11) 0.93 (0.78, 1.10)
Sleep disorders
1.25 (1.15, 1.36) 1.28 (1.19, 1.38)
0.94 (0.86, 1.03) 0.92 (0.83, 1.01)
Headache
1.28 (1.16, 1.40) 1.27 (1.17, 1.37)
1.12 (1.02, 1.22) 1.12 (1.02, 1.22)
Anxiety
1.17 (1.07, 1.27) 1.17 (1.09, 1.26)
0.89 (0.82, 0.97) 0.88 (0.81, 0.96)
Hair loss‡
1.93 (1.67, 2.24) 2.10 (1.84, 2.39)
2.31 (1.88, 2.83) 2.24 (1.82, 2.77)
Pressure ulcers‡
1.92 (1.62, 2.26) 1.96 (1.70, 2.27)
1.76 (1.52, 2.05) 1.75 (1.50, 2.03)
Dermatitis
1.16 (1.05, 1.29) 1.21 (1.10, 1.33)
1.24 (1.10, 1.39) 1.18 (1.05, 1.34)
Paresthesia
1.13 (1.04, 1.22) 1.17 (1.09, 1.26)
1.05 (0.97, 1.15) 1.05 (0.96, 1.15)
Pulmonary fibrosis‡
2.23 (2.01, 2.46) 2.49 (2.29, 2.72)
1.58 (1.42, 1.74) 1.53 (1.38, 1.70)
Dyspnea‡
1.75 (1.66, 1.84) 1.80 (1.72, 1.89)
1.44 (1.35, 1.53) 1.41 (1.33, 1.50)
Acute pharyngitis
1.36 (1.19, 1.57) 1.41 (1.25, 1.60)
1.31 (1.16, 1.47) 1.27 (1.12, 1.44)
COPD
1.27 (1.12, 1.44) 1.29 (1.15, 1.44)
0.94 (0.83, 1.07) 0.93 (0.82, 1.06)
Atelectasis
1.24 (1.11, 1.39) 1.27 (1.15, 1.40)
1.08 (0.98, 1.19) 1.06 (0.96, 1.17)
Pulmonary embolism‡
2.08 (1.78, 2.43) 2.25 (1.96, 2.59)
1.65 (1.41, 1.92) 1.63 (1.39, 1.92)
Thromboembolism
1.57 (1.38, 1.78) 1.64 (1.47, 1.84)
1.05 (0.91, 1.20) 1.03 (0.89, 1.18)
Chest pain‡
1.56 (1.45, 1.67) 1.55 (1.46, 1.66)
1.26 (1.17, 1.36) 1.27 (1.17, 1.37)
Abnormal heartbeat‡
1.38 (1.29, 1.48) 1.40 (1.32, 1.49)
1.19 (1.10, 1.28) 1.17 (1.08, 1.27)
Hypotension
1.32 (1.17, 1.50) 1.34 (1.20, 1.50)
1.08 (0.96, 1.21) 1.05 (0.93, 1.18)
Anemia
1.31 (1.22, 1.41) 1.32 (1.24, 1.41)
1.00 (0.93, 1.08) 0.99 (0.92, 1.07)
Heart failure
1.25 (1.13, 1.39) 1.23 (1.12, 1.35)
0.94 (0.85, 1.05) 0.92 (0.82, 1.02)
Malnutrition
1.50 (1.35, 1.67) 1.57 (1.43, 1.72)
1.05 (0.95, 1.17) 1.04 (0.93, 1.16)
Fluid disorders
1.27 (1.17, 1.37) 1.32 (1.23, 1.41)
1.04 (0.96, 1.12) 1.01 (0.93, 1.09)
Diabetes mellitus
1.26 (1.15, 1.38) 1.27 (1.17, 1.37)
1.11 (1.01, 1.23) 1.11 (1.00, 1.23)
Edema
1.19 (1.12, 1.27) 1.23 (1.16, 1.30)
1.07 (1.00, 1.14) 1.05 (0.99, 1.13)
Constipation
1.22 (1.12, 1.32) 1.19 (1.11, 1.28)
0.92 (0.84, 1.01) 0.91 (0.83, 1.01)
Abdominal pain
1.18 (1.11, 1.25) 1.18 (1.12, 1.24)
0.98 (0.91, 1.04) 0.98 (0.91, 1.04)
Cystitis
1.33 (1.15, 1.54) 1.31 (1.15, 1.49)
1.22 (1.06, 1.41) 1.20 (1.04, 1.40)
Acute kidney failure
1.22 (1.10, 1.34) 1.25 (1.15, 1.36)
1.04 (0.94, 1.14) 1.00 (0.91, 1.10)
Malaise and fatigue‡
1.55 (1.44, 1.67) 1.64 (1.54, 1.75)
1.24 (1.15, 1.33) 1.21 (1.12, 1.31)
Fever
1.35 (1.20, 1.52) 1.49 (1.34, 1.66)
1.14 (1.03, 1.26) 1.12 (1.01, 1.25)
Dizziness
1.21 (1.08, 1.34) 1.24 (1.13, 1.36)
1.05 (0.95, 1.17) 1.04 (0.94, 1.16)
Joint pain
1.15 (1.09, 1.22) 1.18 (1.12, 1.24)
1.08 (1.01, 1.16) 1.08 (1.00, 1.15)
Fibromyalgia
1.13 (1.04, 1.24) 1.18 (1.09, 1.27)
0.93 (0.84, 1.03) 0.92 (0.83, 1.02)
0.7
1
3

Supplementary Fig. 9. The post-acute sequelae of SARS-CoV-2 risks when adjusting for index day through Cubic B-Spline versus Categorical variables, the INSIGHT and OneFlorida+ cohorts, from March 2020 to November 2021. The index days since March 1[st,] 2020, were modeled by a) Cubic B-spline with five knots (namely, 7 spline basis functions of polynomial order 3) and b) categorizing days into five periods (March 2020 – June 2020, July 2020 – October 2020, November 2020 - February 2021, March 2021 – June 2021, July 2021 – November 2021). Adjusted hazard ratios (aHR) were compared when using the cubic B-spline method (aHR spline) versus the categorial method in the primary analysis (aHR category). The PASC conditions identified in both datasets were marked by ‡ symbols. The 95% confidence intervals (CI) of aHR were reported. The color panels represent different organ systems, including (from top to bottom): the nervous system, skin, respiratory system, circulatory system, endocrine and metabolic, digestive system, genitourinary system, and other general signs. The PASC outcomes were ascertained from day 30 after the SARS-CoV-2 infection and all the adjusted risk measures were computed 180 days after the SARS-CoV-2 infection.

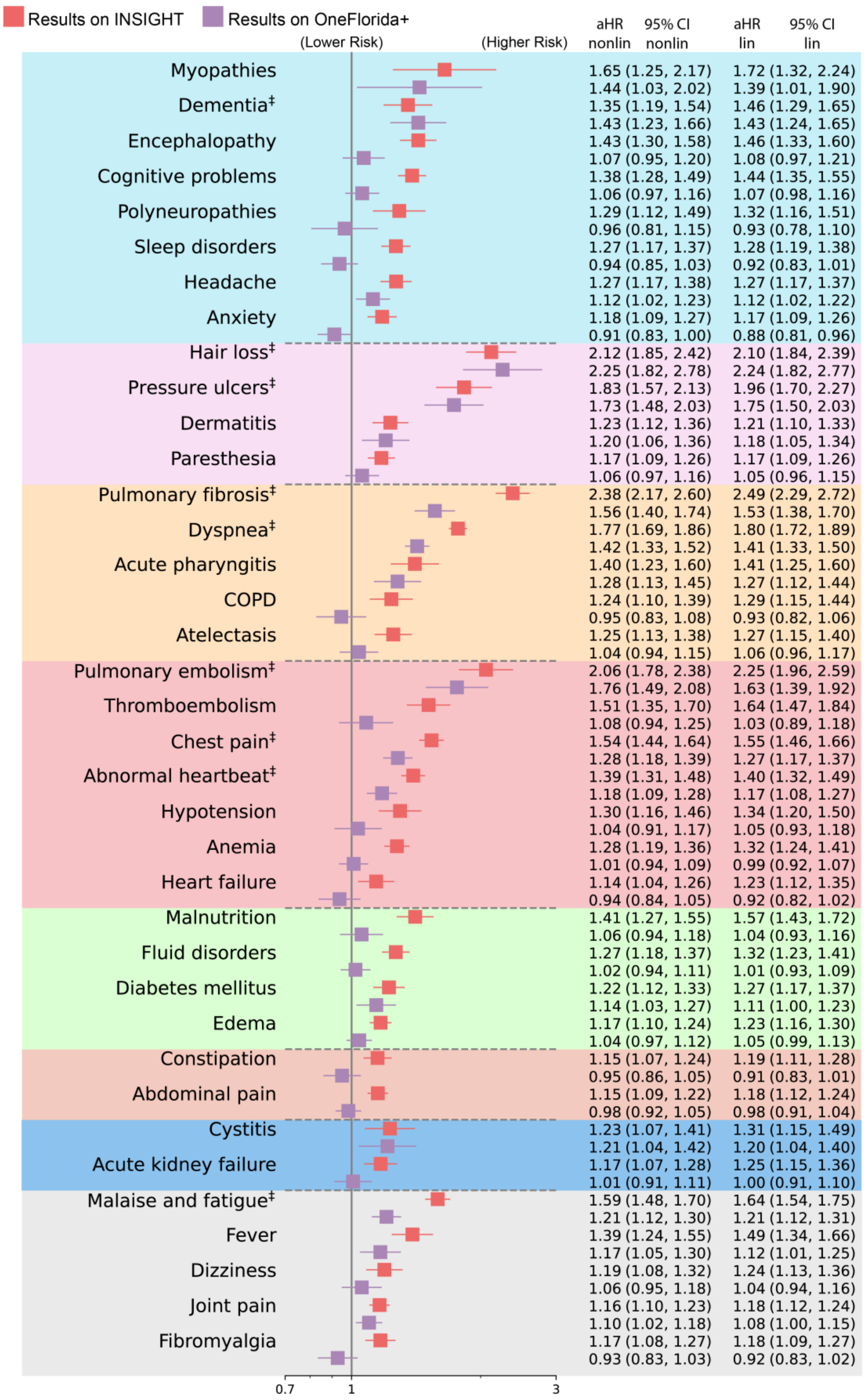

Outcome	Cohort	aHR nonlin	95% CI nonlin	aHR lin	95% CI lin
Myopathies	INSIGHT	1.65	(1.25, 2.17)	1.72	(1.32, 2.24)
	OneFlorida+	1.44	(1.03, 2.02)	1.39	(1.01, 1.90)
Dementia‡	INSIGHT	1.35	(1.19, 1.54)	1.46	(1.29, 1.65)
	OneFlorida+	1.43	(1.23, 1.66)	1.43	(1.24, 1.65)
Encephalopathy	INSIGHT	1.43	(1.30, 1.58)	1.46	(1.33, 1.60)
	OneFlorida+	1.07	(0.95, 1.20)	1.08	(0.97, 1.21)
Cognitive problems	INSIGHT	1.38	(1.28, 1.49)	1.44	(1.35, 1.55)
	OneFlorida+	1.06	(0.97, 1.16)	1.07	(0.98, 1.16)
Polyneuropathies	INSIGHT	1.29	(1.12, 1.49)	1.32	(1.16, 1.51)
	OneFlorida+	0.96	(0.81, 1.15)	0.93	(0.78, 1.10)
Sleep disorders	INSIGHT	1.27	(1.17, 1.37)	1.28	(1.19, 1.38)
	OneFlorida+	0.94	(0.85, 1.03)	0.92	(0.83, 1.01)
Headache	INSIGHT	1.27	(1.17, 1.38)	1.27	(1.17, 1.37)
	OneFlorida+	1.12	(1.02, 1.23)	1.12	(1.02, 1.22)
Anxiety	INSIGHT	1.18	(1.09, 1.27)	1.17	(1.09, 1.26)
	OneFlorida+	0.91	(0.83, 1.00)	0.88	(0.81, 0.96)
Hair loss‡	INSIGHT	2.12	(1.85, 2.42)	2.10	(1.84, 2.39)
	OneFlorida+	2.25	(1.82, 2.78)	2.24	(1.82, 2.77)
Pressure ulcers‡	INSIGHT	1.83	(1.57, 2.13)	1.96	(1.70, 2.27)
	OneFlorida+	1.73	(1.48, 2.03)	1.75	(1.50, 2.03)
Dermatitis	INSIGHT	1.23	(1.12, 1.36)	1.21	(1.10, 1.33)
	OneFlorida+	1.20	(1.06, 1.36)	1.18	(1.05, 1.34)
Paresthesia	INSIGHT	1.17	(1.09, 1.26)	1.17	(1.09, 1.26)
	OneFlorida+	1.06	(0.97, 1.16)	1.05	(0.96, 1.15)
Pulmonary fibrosis‡	INSIGHT	2.38	(2.17, 2.60)	2.49	(2.29, 2.72)
	OneFlorida+	1.56	(1.40, 1.74)	1.53	(1.38, 1.70)
Dyspnea‡	INSIGHT	1.77	(1.69, 1.86)	1.80	(1.72, 1.89)
	OneFlorida+	1.42	(1.33, 1.52)	1.41	(1.33, 1.50)
Acute pharyngitis	INSIGHT	1.40	(1.23, 1.60)	1.41	(1.25, 1.60)
	OneFlorida+	1.28	(1.13, 1.45)	1.27	(1.12, 1.44)
COPD	INSIGHT	1.24	(1.10, 1.39)	1.29	(1.15, 1.44)
	OneFlorida+	0.95	(0.83, 1.08)	0.93	(0.82, 1.06)
Atelectasis	INSIGHT	1.25	(1.13, 1.38)	1.27	(1.15, 1.40)
	OneFlorida+	1.04	(0.94, 1.15)	1.06	(0.96, 1.17)
Pulmonary embolism‡	INSIGHT	2.06	(1.78, 2.38)	2.25	(1.96, 2.59)
	OneFlorida+	1.76	(1.49, 2.08)	1.63	(1.39, 1.92)
Thromboembolism	INSIGHT	1.51	(1.35, 1.70)	1.64	(1.47, 1.84)
	OneFlorida+	1.08	(0.94, 1.25)	1.03	(0.89, 1.18)
Chest pain‡	INSIGHT	1.54	(1.44, 1.64)	1.55	(1.46, 1.66)
	OneFlorida+	1.28	(1.18, 1.39)	1.27	(1.17, 1.37)
Abnormal heartbeat‡	INSIGHT	1.39	(1.31, 1.48)	1.40	(1.32, 1.49)
	OneFlorida+	1.18	(1.09, 1.28)	1.17	(1.08, 1.27)
Hypotension	INSIGHT	1.30	(1.16, 1.46)	1.34	(1.20, 1.50)
	OneFlorida+	1.04	(0.91, 1.17)	1.05	(0.93, 1.18)
Anemia	INSIGHT	1.28	(1.19, 1.36)	1.32	(1.24, 1.41)
	OneFlorida+	1.01	(0.94, 1.09)	0.99	(0.92, 1.07)
Heart failure	INSIGHT	1.14	(1.04, 1.26)	1.23	(1.12, 1.35)
	OneFlorida+	0.94	(0.84, 1.05)	0.92	(0.82, 1.02)
Malnutrition	INSIGHT	1.41	(1.27, 1.55)	1.57	(1.43, 1.72)
	OneFlorida+	1.06	(0.94, 1.18)	1.04	(0.93, 1.16)
Fluid disorders	INSIGHT	1.27	(1.18, 1.37)	1.32	(1.23, 1.41)
	OneFlorida+	1.02	(0.94, 1.11)	1.01	(0.93, 1.09)
Diabetes mellitus	INSIGHT	1.22	(1.12, 1.33)	1.27	(1.17, 1.37)
	OneFlorida+	1.14	(1.03, 1.27)	1.11	(1.00, 1.23)
Edema	INSIGHT	1.17	(1.10, 1.24)	1.23	(1.16, 1.30)
	OneFlorida+	1.04	(0.97, 1.12)	1.05	(0.99, 1.13)
Constipation	INSIGHT	1.15	(1.07, 1.24)	1.19	(1.11, 1.28)
	OneFlorida+	0.95	(0.86, 1.05)	0.91	(0.83, 1.01)
Abdominal pain	INSIGHT	1.15	(1.09, 1.22)	1.18	(1.12, 1.24)
	OneFlorida+	0.98	(0.92, 1.05)	0.98	(0.91, 1.04)
Cystitis	INSIGHT	1.23	(1.07, 1.41)	1.31	(1.15, 1.49)
	OneFlorida+	1.21	(1.04, 1.42)	1.20	(1.04, 1.40)
Acute kidney failure	INSIGHT	1.17	(1.07, 1.28)	1.25	(1.15, 1.36)
	OneFlorida+	1.01	(0.91, 1.11)	1.00	(0.91, 1.10)
Malaise and fatigue‡	INSIGHT	1.59	(1.48, 1.70)	1.64	(1.54, 1.75)
	OneFlorida+	1.21	(1.12, 1.30)	1.21	(1.12, 1.31)
Fever	INSIGHT	1.39	(1.24, 1.55)	1.49	(1.34, 1.66)
	OneFlorida+	1.17	(1.05, 1.30)	1.12	(1.01, 1.25)
Dizziness	INSIGHT	1.19	(1.08, 1.32)	1.24	(1.13, 1.36)
	OneFlorida+	1.06	(0.95, 1.18)	1.04	(0.94, 1.16)
Joint pain	INSIGHT	1.16	(1.10, 1.23)	1.18	(1.12, 1.24)
	OneFlorida+	1.10	(1.02, 1.18)	1.08	(1.00, 1.15)
Fibromyalgia	INSIGHT	1.17	(1.08, 1.27)	1.18	(1.09, 1.27)
	OneFlorida+	0.93	(0.83, 1.03)	0.92	(0.83, 1.02)

Supplementary Fig. 10. The post-acute sequelae of SARS-CoV-2 risks when using non-linear PS modeling versus linear PS modeling, the INSIGHT and OneFlorida+ cohorts, from March 2020 to November 2021. Adjusted hazard ratios (aHR) were adjusted for a nonlinear PS modeling using the gradient boosting machine with decision tree base learners (aHR nonlinear) versus the linear PS modeling using regularized logistic regression in our primary analysis (aHR lin). The PASC conditions identified in both datasets were marked by ‡ symbols. The color panels represent different organ systems, including (from top to bottom): the nervous system, skin, respiratory system, circulatory system, endocrine and metabolic, digestive system, genitourinary system, and other general signs. The PASC outcomes were ascertained from day 30 after the SARS-CoV-2 infection and all the adjusted risk measures were computed 180 days after the SARS-CoV-2 infection. The 95% confidence intervals (CI) of aHR were reported. PS, propensity score.

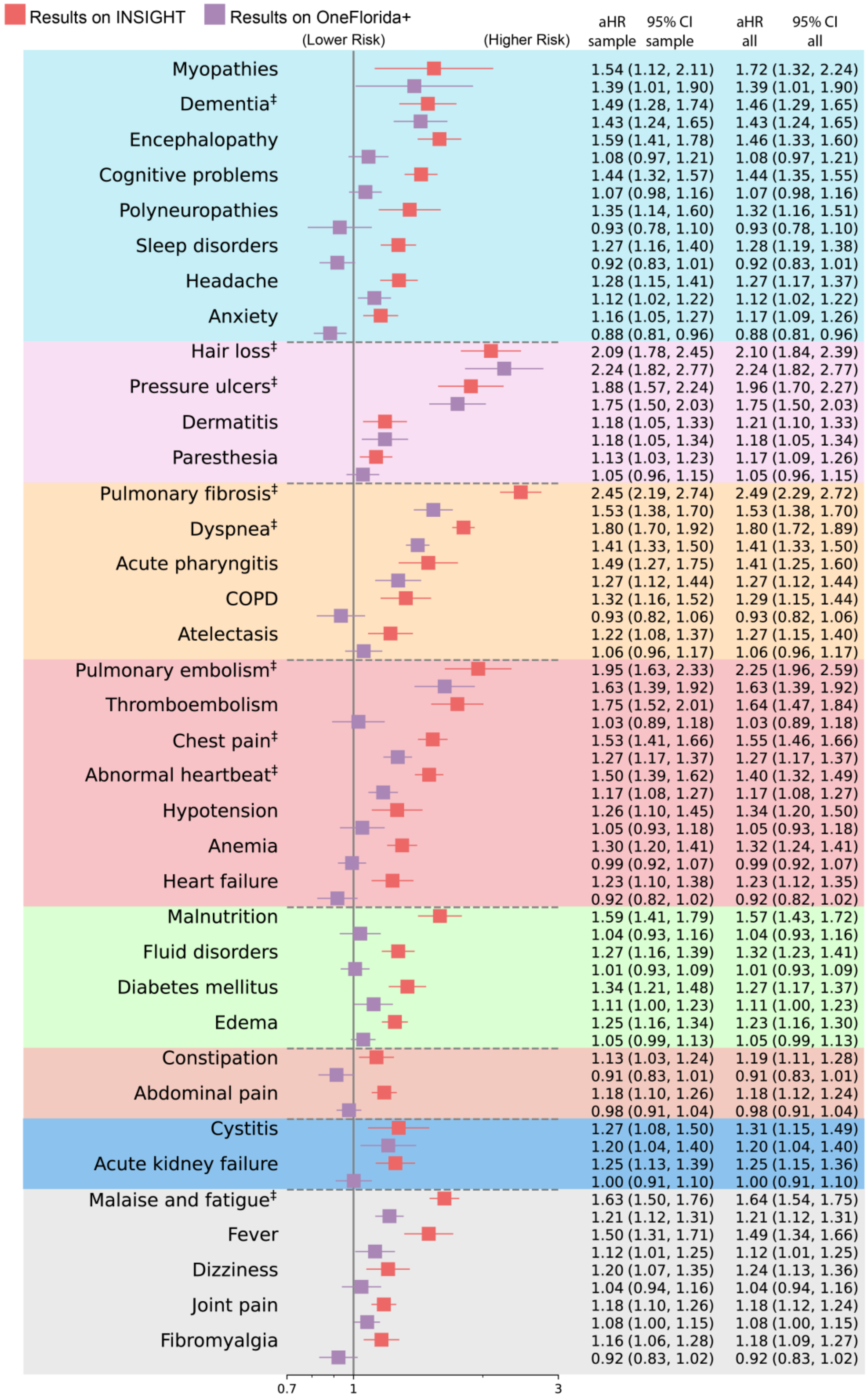

Results on INSIGHT
Results on OneFlorida+
(Lower Risk)
(Higher Risk)
aHR sample
95% CI sample
aHR all
95% CI all
Myopathies
1.54 (1.12, 2.11) 1.72 (1.32, 2.24)
1.39 (1.01, 1.90) 1.39 (1.01, 1.90)
Dementia‡
1.49 (1.28, 1.74) 1.46 (1.29, 1.65)
1.43 (1.24, 1.65) 1.43 (1.24, 1.65)
Encephalopathy
1.59 (1.41, 1.78) 1.46 (1.33, 1.60)
1.08 (0.97, 1.21) 1.08 (0.97, 1.21)
Cognitive problems
1.44 (1.32, 1.57) 1.44 (1.35, 1.55)
1.07 (0.98, 1.16) 1.07 (0.98, 1.16)
Polyneuropathies
1.35 (1.14, 1.60) 1.32 (1.16, 1.51)
0.93 (0.78, 1.10) 0.93 (0.78, 1.10)
Sleep disorders
1.27 (1.16, 1.40) 1.28 (1.19, 1.38)
0.92 (0.83, 1.01) 0.92 (0.83, 1.01)
Headache
1.28 (1.15, 1.41) 1.27 (1.17, 1.37)
1.12 (1.02, 1.22) 1.12 (1.02, 1.22)
Anxiety
1.16 (1.05, 1.27) 1.17 (1.09, 1.26)
0.88 (0.81, 0.96) 0.88 (0.81, 0.96)
Hair loss‡
2.09 (1.78, 2.45) 2.10 (1.84, 2.39)
2.24 (1.82, 2.77) 2.24 (1.82, 2.77)
Pressure ulcers‡
1.88 (1.57, 2.24) 1.96 (1.70, 2.27)
1.75 (1.50, 2.03) 1.75 (1.50, 2.03)
Dermatitis
1.18 (1.05, 1.33) 1.21 (1.10, 1.33)
1.18 (1.05, 1.34) 1.18 (1.05, 1.34)
Paresthesia
1.13 (1.03, 1.23) 1.17 (1.09, 1.26)
1.05 (0.96, 1.15) 1.05 (0.96, 1.15)
Pulmonary fibrosis‡
2.45 (2.19, 2.74) 2.49 (2.29, 2.72)
1.53 (1.38, 1.70) 1.53 (1.38, 1.70)
Dyspnea‡
1.80 (1.70, 1.92) 1.80 (1.72, 1.89)
1.41 (1.33, 1.50) 1.41 (1.33, 1.50)
Acute pharyngitis
1.49 (1.27, 1.75) 1.41 (1.25, 1.60)
1.27 (1.12, 1.44) 1.27 (1.12, 1.44)
COPD
1.32 (1.16, 1.52) 1.29 (1.15, 1.44)
0.93 (0.82, 1.06) 0.93 (0.82, 1.06)
Atelectasis
1.22 (1.08, 1.37) 1.27 (1.15, 1.40)
1.06 (0.96, 1.17) 1.06 (0.96, 1.17)
Pulmonary embolism‡
1.95 (1.63, 2.33) 2.25 (1.96, 2.59)
1.63 (1.39, 1.92) 1.63 (1.39, 1.92)
Thromboembolism
1.75 (1.52, 2.01) 1.64 (1.47, 1.84)
1.03 (0.89, 1.18) 1.03 (0.89, 1.18)
Chest pain‡
1.53 (1.41, 1.66) 1.55 (1.46, 1.66)
1.27 (1.17, 1.37) 1.27 (1.17, 1.37)
Abnormal heartbeat‡
1.50 (1.39, 1.62) 1.40 (1.32, 1.49)
1.17 (1.08, 1.27) 1.17 (1.08, 1.27)
Hypotension
1.26 (1.10, 1.45) 1.34 (1.20, 1.50)
1.05 (0.93, 1.18) 1.05 (0.93, 1.18)
Anemia
1.30 (1.20, 1.41) 1.32 (1.24, 1.41)
0.99 (0.92, 1.07) 0.99 (0.92, 1.07)
Heart failure
1.23 (1.10, 1.38) 1.23 (1.12, 1.35)
0.92 (0.82, 1.02) 0.92 (0.82, 1.02)
Malnutrition
1.59 (1.41, 1.79) 1.57 (1.43, 1.72)
1.04 (0.93, 1.16) 1.04 (0.93, 1.16)
Fluid disorders
1.27 (1.16, 1.39) 1.32 (1.23, 1.41)
1.01 (0.93, 1.09) 1.01 (0.93, 1.09)
Diabetes mellitus
1.34 (1.21, 1.48) 1.27 (1.17, 1.37)
1.11 (1.00, 1.23) 1.11 (1.00, 1.23)
Edema
1.25 (1.16, 1.34) 1.23 (1.16, 1.30)
1.05 (0.99, 1.13) 1.05 (0.99, 1.13)
Constipation
1.13 (1.03, 1.24) 1.19 (1.11, 1.28)
0.91 (0.83, 1.01) 0.91 (0.83, 1.01)
Abdominal pain
1.18 (1.10, 1.26) 1.18 (1.12, 1.24)
0.98 (0.91, 1.04) 0.98 (0.91, 1.04)
Cystitis
1.27 (1.08, 1.50) 1.31 (1.15, 1.49)
1.20 (1.04, 1.40) 1.20 (1.04, 1.40)
Acute kidney failure
1.25 (1.13, 1.39) 1.25 (1.15, 1.36)
1.00 (0.91, 1.10) 1.00 (0.91, 1.10)
Malaise and fatigue‡
1.63 (1.50, 1.76) 1.64 (1.54, 1.75)
1.21 (1.12, 1.31) 1.21 (1.12, 1.31)
Fever
1.50 (1.31, 1.71) 1.49 (1.34, 1.66)
1.12 (1.01, 1.25) 1.12 (1.01, 1.25)
Dizziness
1.20 (1.07, 1.35) 1.24 (1.13, 1.36)
1.04 (0.94, 1.16) 1.04 (0.94, 1.16)
Joint pain
1.18 (1.10, 1.26) 1.18 (1.12, 1.24)
1.08 (1.00, 1.15) 1.08 (1.00, 1.15)
Fibromyalgia
1.16 (1.06, 1.28) 1.18 (1.09, 1.27)
0.92 (0.83, 1.02) 0.92 (0.83, 1.02)
0.7
1
3

Supplementary Fig. 11. The post-acute sequelae of SARS-CoV-2 risks when downsampling the INSIGHT cohort into 22,341 SARS-CoV-2 positive patients and 177,010 negative patients versus using original data, the INSIGHT and OneFlorida+ cohorts, from March 2020 to November 2021. The number of SARS-CoV2 positive (or negative) patients in the INSIGHT after downsampling is the same as the number of positive (or negative) patients in the OneFlorida+. Adjusted hazard ratios (aHR) and 95% confidence intervals (CI) were compared using the downsampled cohorts (aHR sample) versus the original cohorts in our primary analysis (aHR all) for the INSIGHT cohort. The PASC conditions identified in both datasets were marked by ‡ symbols. The color panels represent different organ systems, including (from top to bottom): the nervous system, skin, respiratory system, circulatory system, endocrine and metabolic, digestive system, genitourinary system, and other general signs. The PASC outcomes were ascertained from day 30 after the SARS-CoV-2 infection and all the adjusted risk measures were computed 180 days after the SARS-CoV-2 infection.

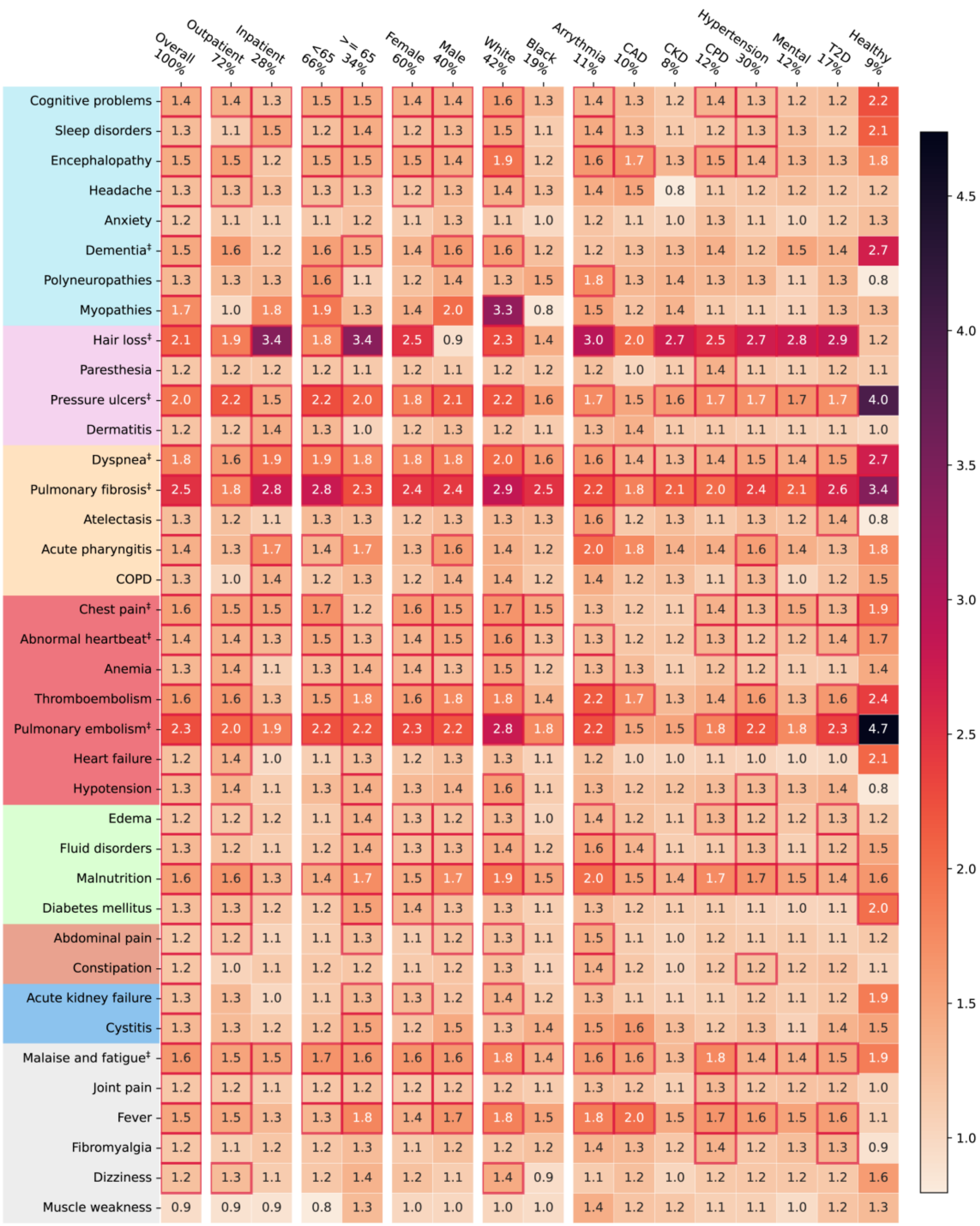

Overall 100%
Outpatient 72%
Inpatient 28%
<65 66%
>= 65 34%
Female 60%
Male 40%
White 42%
Black 19%
Arrythmia 11%
CAD 10%
CKD 8%
CPD 12%
Hypertension 30%
Mental 12%
T2D 17%
Healthy 9%
Cognitive problems 1.4 1.4 1.3 1.5 1.5 1.4 1.4 1.6 1.3 1.4 1.3 1.2 1.4 1.3 1.2 1.2 2.2
Sleep disorders 1.3 1.1 1.5 1.2 1.4 1.2 1.3 1.5 1.1 1.4 1.3 1.1 1.2 1.3 1.3 1.2 2.1
Encephalopathy 1.5 1.5 1.2 1.5 1.5 1.5 1.4 1.9 1.2 1.6 1.7 1.3 1.5 1.4 1.3 1.3 1.8
Headache 1.3 1.3 1.3 1.3 1.3 1.2 1.3 1.4 1.3 1.4 1.5 0.8 1.1 1.2 1.2 1.2 1.2
Anxiety 1.2 1.1 1.1 1.1 1.2 1.1 1.3 1.1 1.0 1.2 1.1 1.0 1.3 1.1 1.0 1.2 1.3
Dementia‡ 1.5 1.6 1.2 1.6 1.5 1.4 1.6 1.6 1.2 1.2 1.3 1.3 1.4 1.2 1.5 1.4 2.7
Polyneuropathies 1.3 1.3 1.3 1.6 1.1 1.2 1.4 1.3 1.5 1.8 1.3 1.4 1.3 1.3 1.1 1.3 0.8
Myopathies 1.7 1.0 1.8 1.9 1.3 1.4 2.0 3.3 0.8 1.5 1.2 1.4 1.1 1.1 1.1 1.3 1.3
Hair loss‡ 2.1 1.9 3.4 1.8 3.4 2.5 0.9 2.3 1.4 3.0 2.0 2.7 2.5 2.7 2.8 2.9 1.2
Paresthesia 1.2 1.2 1.2 1.2 1.1 1.2 1.1 1.2 1.2 1.2 1.0 1.1 1.4 1.1 1.1 1.2 1.1
Pressure ulcers‡ 2.0 2.2 1.5 2.2 2.0 1.8 2.1 2.2 1.6 1.7 1.5 1.6 1.7 1.7 1.7 1.7 4.0
Dermatitis 1.2 1.2 1.4 1.3 1.0 1.2 1.3 1.2 1.1 1.3 1.4 1.1 1.1 1.1 1.1 1.1 1.0
Dyspnea‡ 1.8 1.6 1.9 1.9 1.8 1.8 1.8 2.0 1.6 1.6 1.4 1.3 1.4 1.5 1.4 1.5 2.7
Pulmonary fibrosis‡ 2.5 1.8 2.8 2.8 2.3 2.4 2.4 2.9 2.5 2.2 1.8 2.1 2.0 2.4 2.1 2.6 3.4
Atelectasis 1.3 1.2 1.1 1.3 1.3 1.2 1.3 1.3 1.3 1.6 1.2 1.3 1.1 1.3 1.2 1.4 0.8
Acute pharyngitis 1.4 1.3 1.7 1.4 1.7 1.3 1.6 1.4 1.2 2.0 1.8 1.4 1.4 1.6 1.4 1.3 1.8
COPD 1.3 1.0 1.4 1.2 1.3 1.2 1.4 1.4 1.2 1.4 1.2 1.3 1.1 1.3 1.0 1.2 1.5
Chest pain‡ 1.6 1.5 1.5 1.7 1.2 1.6 1.5 1.7 1.5 1.3 1.2 1.1 1.4 1.3 1.5 1.3 1.9
Abnormal heartbeat‡ 1.4 1.4 1.3 1.5 1.3 1.4 1.5 1.6 1.3 1.3 1.2 1.2 1.3 1.2 1.2 1.4 1.7
Anemia 1.3 1.4 1.1 1.3 1.4 1.4 1.3 1.5 1.2 1.3 1.3 1.1 1.2 1.2 1.1 1.1 1.4
Thromboembolism 1.6 1.6 1.3 1.5 1.8 1.6 1.8 1.8 1.4 2.2 1.7 1.3 1.4 1.6 1.3 1.6 2.4
Pulmonary embolism‡ 2.3 2.0 1.9 2.2 2.2 2.3 2.2 2.8 1.8 2.2 1.5 1.5 1.8 2.2 1.8 2.3 4.7
Heart failure 1.2 1.4 1.0 1.1 1.3 1.2 1.3 1.3 1.1 1.2 1.0 1.0 1.1 1.0 1.0 1.0 2.1
Hypotension 1.3 1.4 1.1 1.3 1.4 1.3 1.4 1.6 1.1 1.3 1.2 1.2 1.3 1.3 1.3 1.4 0.8
Edema 1.2 1.2 1.2 1.1 1.4 1.3 1.2 1.3 1.0 1.4 1.2 1.1 1.3 1.2 1.2 1.3 1.2
Fluid disorders 1.3 1.2 1.1 1.2 1.4 1.3 1.3 1.4 1.2 1.6 1.4 1.1 1.1 1.3 1.1 1.2 1.5
Malnutrition 1.6 1.6 1.3 1.4 1.7 1.5 1.7 1.9 1.5 2.0 1.5 1.4 1.7 1.7 1.5 1.4 1.6
Diabetes mellitus 1.3 1.3 1.2 1.2 1.5 1.4 1.3 1.3 1.1 1.3 1.2 1.1 1.1 1.1 1.0 1.1 2.0
Abdominal pain 1.2 1.2 1.1 1.1 1.3 1.1 1.2 1.3 1.1 1.5 1.1 1.0 1.2 1.1 1.1 1.1 1.2
Constipation 1.2 1.0 1.1 1.2 1.2 1.1 1.2 1.3 1.1 1.4 1.2 1.0 1.2 1.2 1.2 1.2 1.1
Acute kidney failure 1.3 1.3 1.0 1.1 1.3 1.3 1.2 1.4 1.2 1.3 1.1 1.1 1.1 1.2 1.1 1.2 1.9
Cystitis 1.3 1.3 1.2 1.2 1.5 1.2 1.5 1.3 1.4 1.5 1.6 1.3 1.2 1.3 1.1 1.4 1.5
Malaise and fatigue‡ 1.6 1.5 1.5 1.7 1.6 1.6 1.6 1.8 1.4 1.6 1.6 1.3 1.8 1.4 1.4 1.5 1.9
Joint pain 1.2 1.2 1.1 1.2 1.2 1.2 1.2 1.2 1.1 1.3 1.2 1.1 1.3 1.2 1.2 1.2 1.0
Fever 1.5 1.5 1.3 1.3 1.8 1.4 1.7 1.8 1.5 1.8 2.0 1.5 1.7 1.6 1.5 1.6 1.1
Fibromyalgia 1.2 1.1 1.2 1.2 1.3 1.1 1.2 1.2 1.2 1.4 1.3 1.2 1.4 1.2 1.3 1.3 0.9
Dizziness 1.2 1.3 1.1 1.2 1.4 1.2 1.1 1.4 0.9 1.1 1.2 1.0 1.2 1.2 1.2 1.2 1.6
Muscle weakness 0.9 0.9 0.9 0.8 1.3 1.0 1.0 1.0 1.0 1.4 1.2 1.2 1.1 1.1 1.0 1.2 1.3
4.5
4.0
3.5
3.0
2.5
2.0
1.5
1.0

Supplementary Fig. 12. Adjusted hazard ratios of post-acute sequelae of SARS-CoV-2 infection (PASC) over different subgroups, the INSIGHT cohort, from March 2020 to November 2021. Subgroups were stratified by their acute severity status, age groups, gender, race groups, and baseline pre-existing conditions. Different color panels represent different organ systems, including (from top to bottom): the nervous system, skin, respiratory system, circulatory system, blood-forming organs, endocrine and metabolic, digestive system, genitourinary system, and general signs. CAD, coronary artery disease; CKD, chronic kidney disease; CPD, chronic pulmonary disease; T2D, diabetes type 2; Healthy: no documented pre-existing conditions and no PASC-like symptoms at baseline. The PASC conditions also identified in OneFlorida+ were marked by ‡ symbols. The conditions with their aHRs' P-value$< 8.39 \times 10^{-5}$(the Bonferroni-corrected significance threshold) were highlighted in red squares. The fraction of the subgroup population was shown at the top.

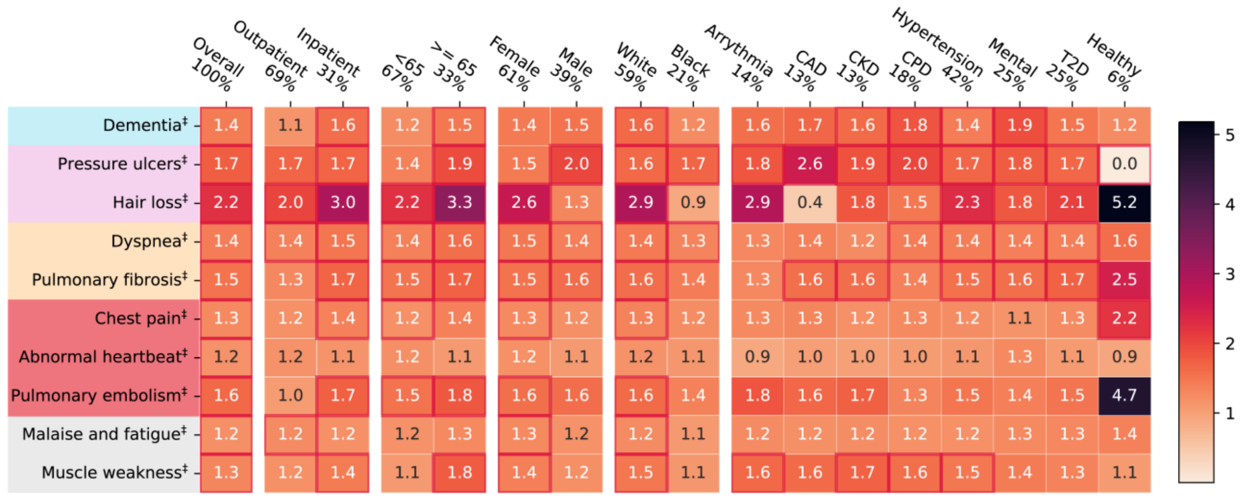

Supplementary Fig. 13. Adjusted hazard ratios of post-acute sequelae of SARS-CoV-2 infection (PASC) over different subgroups, the OneFlorida+ cohort, from March 2020 to November 2021. Subgroups were stratified by their acute severity status, age groups, gender, race groups, and baseline pre-existing conditions. Different color panels represent different organ systems, including (from top to bottom): the nervous system, skin, respiratory system, circulatory system, and general signs. CAD, coronary artery disease; CKD, chronic kidney disease; CPD, chronic pulmonary disease; T2D, diabetes type 2; Healthy: no documented pre-existing conditions and no PASC-like symptoms at baseline. The conditions with their aHRs' P-value $< 8.39 \times 10^{-5}$(the Bonferroni-corrected significance threshold) were highlighted in red squares. The fraction of the subgroup population was shown at the top.

Supplementary Table 1. Results of negative outcome control in both the INSIGHT and OneFlorida+ cohorts, March 2020–November 2021.

Results of negative outcome control in both the INSIGHT and OneFlorida+ cohorts, March 2020–November 2021.

Negative Outcomes	Adjusted Hazard Ratio (95% Confidence Interval)[a]	aHR's P-value[b]	No. of SARS-CoV-2 Positive patients	No. of SARS-CoV-2 Negative patients [c]	Number of events in the case group	Number of events in the control group	No. of unbalanced covariates	No. of unbalanced covariates after re-weighting[a]
**INSIGHT**								
Accidental Injuries	1.02 (0.84, 1.23)	0.87	33,898	33,898	347	248	31	0
Benign Neoplasms	0.93 (0.84, 1.03)	0.14	30,461	30,461	1030	1228	31	0
**OneFlorida+**								
Accidental Injuries	0.99 (0.88, 1.12)	0.92	18,967	18967	661	599	22	0
Benign neoplasms	0.93 (0.82, 1.05)	0.23	18,729	18729	585	693	21	0

a. Outcomes were ascertained from day 30 after the SARS-CoV-2 infection. The adjusted hazard ratios were computed 180 days after the SARS-CoV-2 infection by adjusting high-dimensional baseline variables the same as in the screening sequelae using inverse probability of treatment weighting as discussed in the Method section. All the baseline covariates were balanced in terms of standardized mean difference < 0.1.

b. An aHR close to 1 with a large P-value (> 0.05) indicates no significant association between SARS-CoV-2 infection and outcomes was found. The aHR and its P-value were calculated by the Cox proportional hazard model and the Wald Chi-Square test.

c. The number of patients in the control group was randomly selected from all SARS-CoV-2 negative patients with the same number as patients in the case group.

Supplementary Table 2. Specifications of the high-throughput screening framework for identifying potential Post-Acute Sequelae of SARS-CoV-2 infected (PASC) using the INSIGHT Electronic Health Records in New York City and OneFlorida+ in Florida (March 2020 – November 2021).

**Protocol component**	**Specifications of hypothetical target trials**	**High-throughput emulation**
Eligibility criteria	• Age ≥20 years at index, and no upper age limit, between March 1, 2020, and November 30, 2021.<br>• Patients without any positive SARS-CoV-2 polymerase-chain-reaction (PCR) or Antigen test, or COVID-19 diagnoses before the index<br>• Use of the INSIGHT health care system, defined as at least one encounter with any diagnoses, in the past 3 years to 7 days before the index.<br>• Use of the INSIGHT health care system, defined as at least one encounter with any diagnoses, 31 days to 180 days after index in the follow-up period, indicates being alive after the potential COVID-19 acute phase and at least 31 days of potential follow-up.<br>• Known documented SARS-CoV-2 PCR/Antigen lab positive or negative results<br>• (High-throughput scenario for PASC) No history of target post-acute sequelae of COVID-19 in the past 3 years to 7 days before the index.<br>• (High-throughput scenario for PASC medications) No history of target medication usage in the past 1 year to 7 days before the index.	Same as for the hypothetical target trials. We identified the SARS-CoV-2 infected PCR/Antigen tests using the laboratory results table, and the COVID-19 diagnoses using ICD-10 codes in the patients' diagnosis table in the INSIGHT EHR system or OneFlorida+ EHR system, following the PCORnet data model

Exposure strategies	• Exposure group: Infection of SARS-CoV-2 and the SARS-CoV-2 PCR/Antigen tested positive<br>• Control group: No infection of SARS-CoV-2, and the SARS-CoV-2 PCR/Antigen tests kept negative in the follow-up period	• Same as for the hypothetical target trial.<br>• SARS-CoV-2 infected cases were defined as patients who had any SARS-CoV-2 positive PCR/Antigen test, and the baseline date, or the index date, is defined as the date of the first documented positive SARS-CoV-2 PCR/Antigen test.<br>• Non-infected controls were defined as patients whose SARS-CoV-2 PCR/Antigen tests were all negative, and there were no COVID-19-related diagnoses documented at any time. The baseline date, or the index date, is defined as the date of the first documented SARS-CoV-2 PCR/Antigen test.
Group assignment	Individuals are randomly assigned to an exposure strategy at baseline and are aware of the assigned exposure strategy.	We classified patients into different exposure groups according to their baseline eligibility criteria and exposure strategies. We assumed that the exposure group and the control group were exchangeable by adjusting for baseline covariates, including age, gender, race, ethnicity, social-economic status, hospital utilization history, period of infection, baseline comorbidities, history of prescriptions, etc.
High-throughput Outcomes	• 137 potential post-acute sequelae of COVID-19 (PASC)<br>• 459 categories of drugs due to post-acute sequelae of COVID-19<br>Hospitalization due to PASC<br>ICU admission due to PASC<br>Death due to PASC (defined as death after 31 days of SARS-CoV-2 infection)	Same as for the hypothetical target trials.
Follow-up	We followed each patient from his/her baseline day until the day of the outcome of interest, death, 180 days after baseline, or the end of the study period (November 30, 2021), whichever happens first.	Same as for the hypothetical target trial.

Outcome contrasts	Excess risk of newly-onset post-acute sequelae of COVID-19 against baseline incidence. The PASC outcomes were ascertained from day 30 after the SARS-CoV-2 infection and all the adjusted risk measures were computed 180 days after the SARS-CoV-2 infection.	Same as for the hypothetical target trial.
High-throughput trials for screening PASC	For each target PASC outcome, we conducted a corresponding hypothetical target trial among which patients were free of the target PASC outcome at baseline and had no history of target outcome before baseline. The number of potential PASCs is large and thus the number of trials is large.	We emulated 137 trials for potential PASC diagnosis outcomes and 459 trials for potential PASC medication outcomes. For each emulated trial, the exposure group consisted of eligible SARS-CoV-2 infected patients without a history of target outcome at baseline. The control group was built from SARS-CoV-2-negative patients who were also without any history of target outcome at baseline. The ratio of the number of patients in the exposure group and the control group was 1:5.
Statistical analysis	Cumulative incidence (risk) curves, excess burden, and hazard ratio (HR) between the two exposure groups were estimated at 6 months.<br>Subgroup analyses by baseline age, race, gender, and severity of acute phase of COVID-19, and different waves. Sensitivity analyses.	Same as for the hypothetical target trial.<br>• We used inverse probability of treatment weighting (IPTW) to adjust for high-dimensional baseline covariates. We used L2-norm logistic regression for propensity score calculation, and the best PS model was selected in 10-fold cross-validations for both goodness-of-balance and goodness-of-fit.<br>• Adjusted Cox proportional hazard model, Kaplan-Meier estimator, and Aalen-Johansen estimator were used.<br>• The p-value was corrected by the Bonferroni method for multiple tests of diagnoses and medications separately.<br>• Potential PASCs were selected according to the number of patients with target outcome in the real-world data (>100), the corrected P-value (<0.05/596), and the hazard ratio (>1)

Abbreviations: PCORnet, the National Patient-Centered Clinical Research Network; PASC, Post-Acute Sequelae of COVID-19; PCR, polymerase chain reaction; aHR, adjusted hazard ratio; IPTW, inverse probability of treatment weighting

Supplementary Table 3. Cross-validation algorithm tailored for our machine learning-based propensity score calculation for each emulated trial.

Table S2. Cross-validation algorithm for the ML-based PS model training, selection, and evaluation

Input:

$(\mathbf{X}, \mathbf{T})$: n patients' covariates and exposure assignment where $\mathbf{T} \in \{0, 1\}^n$;

$\mathbf{F}_{\mathbf{\Theta}}$: a set of machine learning-based propensity score (PS) models;

Output:

f_{best}: the best PS model learned from $(\mathbf{X}, \mathbf{T})$

Goodness-of-balance performance, and goodness-of-fit performance

1. **for** each f_θ in $\mathbf{F}_{\mathbf{\Theta}}$ **do**:
2. randomly splitting $(\mathbf{X}, \mathbf{T})$ into K ($K = 10$ in our experiments) equal-sized subsets
3. **for** each $(\mathbf{X}_i, \mathbf{T}_i)$ subset in the K subsets **do**:
4. training f_θ on the remaining $K - 1$ subsets $(\boldsymbol{X}_{K-i}, \mathbf{T}_{K-i})$ by optimizing binary cross entropy loss $L(\mathbf{T}, f_\theta(\mathbf{X}))$
5. computing trimmed and stabilized IPTW $\mathbf{w}$ by using f_θ on $(\mathbf{X}, \mathbf{T})$
6. computing re-weighted SMD_i on $(\mathbf{X}, \mathbf{T})$ by using $\mathbf{w}$
7. computing the number of unbalanced features $n_{\text{unbalance}-i}$ after IPTW
8. computing the AUC_i of f_θ on the testing set $(\mathbf{X}_i, \mathbf{T}_i)$
9. computing f_θ's average goodness-of-balance performance $n_{\text{unbalance}-\theta} = E_{i\sim K}[n_{\text{unbalance}-i}]$ and goodness-of-fit performance $\text{AUC}_\theta = E_{i\sim K}[\text{AUC}_i]$ over K folds
10. updating best-selected model $f_{\text{best}} := f_\theta$, the best performance $n_{\text{unbalance}-\text{best}}$ and AUC_{best} if $n_{\text{unbalance}-\theta}$ is smaller than the current minimum $n_{\text{unbalance}-\text{best}}$, or $n_{\text{unbalance}-\theta}$ is equal to the current minimum $n_{\text{unbalance}-\text{best}}$ but the AUC_θ is larger than the current maximum AUC_{best}
11. re-training f_{best} on the whole dataset $(\mathbf{X}, \mathbf{T})$
12. re-computing stabilized IPTW $\mathbf{w}_{\text{best}}$ by using learned f_{best} on $(\mathbf{X}, \mathbf{T})$
13. re-computing re-weighted SMD_{best} on $(\mathbf{X}, \mathbf{T})$ by using $\boldsymbol{w}_{\text{best}}$
14. re-computing the number of unbalanced features $n_{\text{unbalance}-\text{best}}$ after IPTW
15. **return** f_{best}, $n_{\text{unbalance}-\text{best}}$ and AUC_{best}

Supplementary Table 4. Baseline SARS-CoV-2 vaccination status recorded in the two EHR databases[a]

INSIGHT (March 2020 - November 2021)						
	Overall	%	SARS-CoV-2 Positive	%	SARS-CoV-2 Negative	%
N	361401	100.0%	35275	100.0%	326126	100.0%
Fully vaccinated[b]	8293	2.3%	358	1.0%	7935	2.4%
Partially vaccinated[c]	6703	1.9%	474	1.3%	6229	1.9%
No evidence	346406	95.9%	34444	97.6%	311962	95.7%
**INSIGHT (December 2020 - November 2021)[d]**						
N	154676	100.0%	20301	100.0%	134375	100.0%
Fully vaccinated[b]	8293	5.4%	358	1.8%	7935	5.9%
Partially vaccinated[c]	6699	4.3%	474	2.3%	6225	4.6%
No evidence	139685	90.3%	19470	95.9%	120215	89.5%
**OneFlorida+ (March 2020 - November 2021)**						
	Overall	%	SARS-CoV-2 Positive	%	SARS-CoV-2 Negative	%
N	199351	100.0%	22341	100.0%	177010	100.0%
Fully vaccinated[b]	1161	0.6%	98	0.4%	1063	0.6%
Partially vaccinated[c]	499	0.3%	67	0.3%	432	0.2%
No evidence	197691	99.2%	22176	99.3%	175515	99.2%
**OneFlorida+ (December 2020 - November 2021)[d]**						
N	88021	100.0%	13074	100.0%	74947	100.0%
Fully vaccinated[b]	1161	1.3%	98	0.7%	1063	1.4%
Partially vaccinated[c]	499	0.6%	67	0.5%	432	0.6%
No evidence	86361	98.1%	12909	98.7%	73452	98.0%

a. Vaccination capture at baseline. b.The fully vaccinated status is defined as two shots of mRNA vaccine (Pfizer, or Moderna) or one shot of J&J before baseline according to https://www.cdc.gov/coronavirus/2019-ncov/vaccines/stay-up-to-date.html. c. The partially vaccinated status is defined as having any recorded vaccination events but not meeting the fully vaccinated criteria. d. The earliest available vaccine began in early December 2020, and nearly half of the study patients got infected before the vaccine was available. Besides, only 9.7% population in INSIGHT had any baseline vaccination capture after December 1st, 2020, and 1.8% population for OneFlorida+

Supplementary Data 1. COVID-19 Phenotyping Lab LOINC codes and Diagnosis ICD10 codes (spreadsheet).

Supplementary Data 2. PASC Adult Diagnostic List for Screening (spreadsheet).

Supplementary Data 3. Baseline population characteristics with more comorbidities information, INSIGHT and OneFlorida+ cohorts, March 2020 to November 2021 (spreadsheet).

Supplementary Data 4. Characteristics of PASC-Specific Cohorts on INSIGHT, NYC, March 2020 to November 2021 (spreadsheet).

Supplementary Data 5. Characteristics of PASC-Specific Cohorts on OneFlorida, Florida, March 2020 to November 2021 (spreadsheet).
