# Supplementary material for: Data-driven analysis to understand long COVID using electronic health records from the RECOVER initiative
Source: Nat Commun. 2023 Apr 7;14:1948. doi: 10.1038/s41467-023-37653-z (PMC10080528; doi:10.1038/s41467-023-37653-z)
Supplement: Supplementary file 3 — Description of Additional Supplementary Files [file 41467_2023_37653_MOESM3_ESM.pdf]

Description of Additional Supplementary Files

File Name: Supplementary Data 1
Description: COVID-19 Phenotyping Lab LOINC codes and Diagnosis ICD10 codes

File Name: Supplementary Data 2
Description: PASC Adult Diagnostic List for Screening

File Name: Supplementary Data 3
Description: Baseline population characteristics with more comorbidities information, INSIGHT and OneFlorida+ cohorts, March 2020 to November 2021

File Name: Supplementary Data 4
Description: Characteristics of PASC-Specific Cohorts on INSIGHT, NYC, March 2020 to November 2021

File Name: Supplementary Data 5
Description: Characteristics of PASC-Specific Cohorts on OneFlorida, Florida, March 2020 to November 2021
